# Altered non-coding RNA expression profile in F_1_ progeny 1 year after parental irradiation is linked to adverse effects in zebrafish

**DOI:** 10.1038/s41598-021-83345-3

**Published:** 2021-02-18

**Authors:** Leonardo Martín, Jorke H. Kamstra, Selma Hurem, Leif C. Lindeman, Dag A. Brede, Håvard Aanes, Igor Babiak, Amilcar Arenal, Deborah Oughton, Brit Salbu, Jan Ludvig Lyche, Peter Aleström

**Affiliations:** 1 Morphophysiology Department, Faculty of Agricultural Sciences, University of Camagüey Ignacio Agramonte y Loynaz, 74 650 Camagüey, Cuba; 2 CERAD CoE, Department of Paraclinical Sciences, Norwegian University of Life Sciences, P.O. Box 5003, Ås, Norway; 3 Institute for Risk Assessment Sciences (IRAS), Utrecht University, Utrecht, The Netherlands; 4 Department of Paraclinical Sciences, Norwegian University of Life Sciences, 0454 Oslo, Norway; 5 Department of Preclinical Sciences and Pathology, Norwegian University of Life Sciences, 0454 Oslo, Norway; 6 Department of Environmental Science, Norwegian University of Life Sciences, 1433 Ås, Norway; 7 PatoGen AS, P.O.box 548, 6001 Ålesund, Norway; 8 Faculty of Biosciences and Aquaculture, Nord University, 8026 Bodø, Norway

**Keywords:** High-throughput screening, Risk factors, Natural hazards

## Abstract

Gamma radiation produces DNA instability and impaired phenotype. Previously, we observed negative effects on phenotype, DNA methylation, and gene expression profiles, in offspring of zebrafish exposed to gamma radiation during gametogenesis. We hypothesize that previously observed effects are accompanied with changes in the expression profile of non-coding RNAs, inherited by next generations. Non-coding RNA expression profile was analysed in F_1_ offspring (5.5 h post-fertilization) by high-throughput sequencing 1 year after parental irradiation (8.7 mGy/h, 5.2 Gy total dose). Using our previous F_1_-γ genome-wide gene expression data (GSE98539), hundreds of mRNAs were predicted as targets of differentially expressed (DE) miRNAs, involved in pathways such as insulin receptor, NFkB and PTEN signalling, linking to apoptosis and cancer. snRNAs belonging to the five major spliceosomal snRNAs were down-regulated in the F_1_-γ group, Indicating transcriptional and post-transcriptional alterations. In addition, DEpiRNA clusters were associated to 9 transposable elements (TEs) (LTR, LINE, and TIR) (p = 0.0024), probable as a response to the activation of these TEs. Moreover, the expression of the lincRNAs *malat-1*, and several others was altered in the offspring F_1_, in concordance with previously observed phenotypical alterations. In conclusion, our results demonstrate diverse gamma radiation-induced alterations in the ncRNA profiles of F_1_ offspring observable 1 year after parental irradiation.

## Introduction

Ionizing radiation can induce direct or indirect DNA damages, causing single or double-stranded breaks, or ionization of water resulting in the formation of free radicals. As a result, the cell responds through DNA damage detection, signalling and repair, or apoptosis and cell death^1^. The cell response cascade to gamma radiation includes changes in gene expression through epigenetic modifications such as post-translational histone modifications, DNA methylation, and microRNAs (miRNAs) such as the oncomir miR-21^2–4^.

Recent studies have shown that zebrafish embryos are affected by ionizing radiation at the mRNA transcriptional level, generating impaired phenotypes, such as higher mortality rate, delayed hatching, altered embryo length, and malformations^5–7^. However, besides miRNAs, for several other classes of small non-coding RNAs (sncRNAs) such as small interfering RNA (siRNA), PIWI-interacting RNA (piRNA), small nuclear RNA (snRNA), and small nucleolar RNA (snoRNA), which have important roles in controlling gene expression, their expression profiles, as well as their contribution to the establishment of gene expression patterns, disorders, and phenotypes under the influence of radiation, remain unknown.

Gamma radiation-induced alterations at transcriptional, and DNA level can be inherited by the offspring of vertebrates. We recently demonstrated the inheritance of altered mRNA expression profiles, DNA methylation, and histone modifications patterns in zebrafish embryos after parental gamma irradiation^8–10^. The DNA methylation analysis along with the mRNA expression profile of F_1_ embryos revealed pathways associated with gamma radiation response; such as molecular mechanisms of cancer, DNA damage response and cell death, along with pathways not previously seem involved in the response to gamma radiation, like signalling and retinoic acid receptor activation, and gonadotropin-releasing hormone (Gnrh) signalling. On the other hand, F_1_ embryos showed enriched methylation at histone marks such as H3K4me3, H3K4me9, and H3K27me3, indicating alterations in chromatin structure and organization, as well as in the expression of developmental genes like hepatocyte nuclear factor 4 alpha (*hnf4a*)^8–10^.

Nevertheless, the inheritance of dysregulated profiles for most of the sncRNA classes in zebrafish offspring, as well as their involvement in the mechanisms underlying the response to gamma radiation, as a result of parental exposure, remains unclear.

Moreover, our previously published work on mRNA expression profile after parental gamma irradiation, using the same sample materials as for the current study, showed genes such as *dicer*, *ago1*, *ago2*, *ago3b*, *ago4*, and *piwil2* to be down-regulated in F_1_ offspring of zebrafish^9^. The products of these genes participate in the miRNA, siRNA, and piRNA biogenesis pathways, suggesting a potential impact of gamma radiation on their biogenesis and expression in the progeny of gamma-exposed parents.

In the present study, we aimed to investigate the effect of gamma radiation on the sncRNA expression profile in F_1_ embryos 1 year after parental exposure through small RNA sequencing and subsequent gene expression analysis.

## Results and discussion

### Sequencing analysis

The sncRNA transcriptome in zebrafish embryos has previously been characterized and consist of several classes of small RNAs such as tRNA-halves, tRNA fragments, piRNAs, and miRNAs among others^11–14^. In this study, we have focused on sncRNA expression through small RNA-seq to analyse the sncRNA profile in 5.5 hpf F_1_ offspring embryos 1 year after parental exposure to gamma radiation (8.7 mGy/h) during gametogenesis.

**Figure 1:**
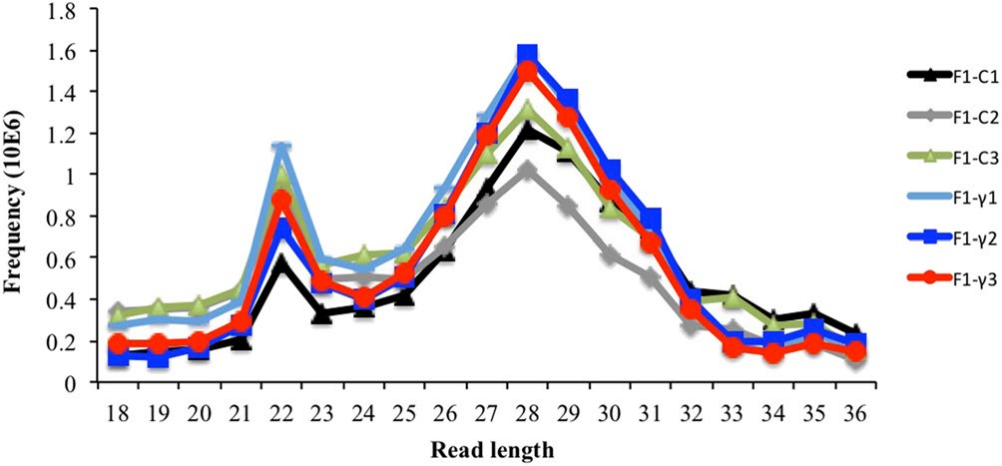
Reads length distribution of raw sequence reads after filtering by read length. Three biological replicate libraries per group of F_1_ generation (5.5 hpf embryos) from parents exposed to 8.7 mGy/h of γ-radiation (F_1_-γ) and control parents (F_1_-C).

**Figure 2:**
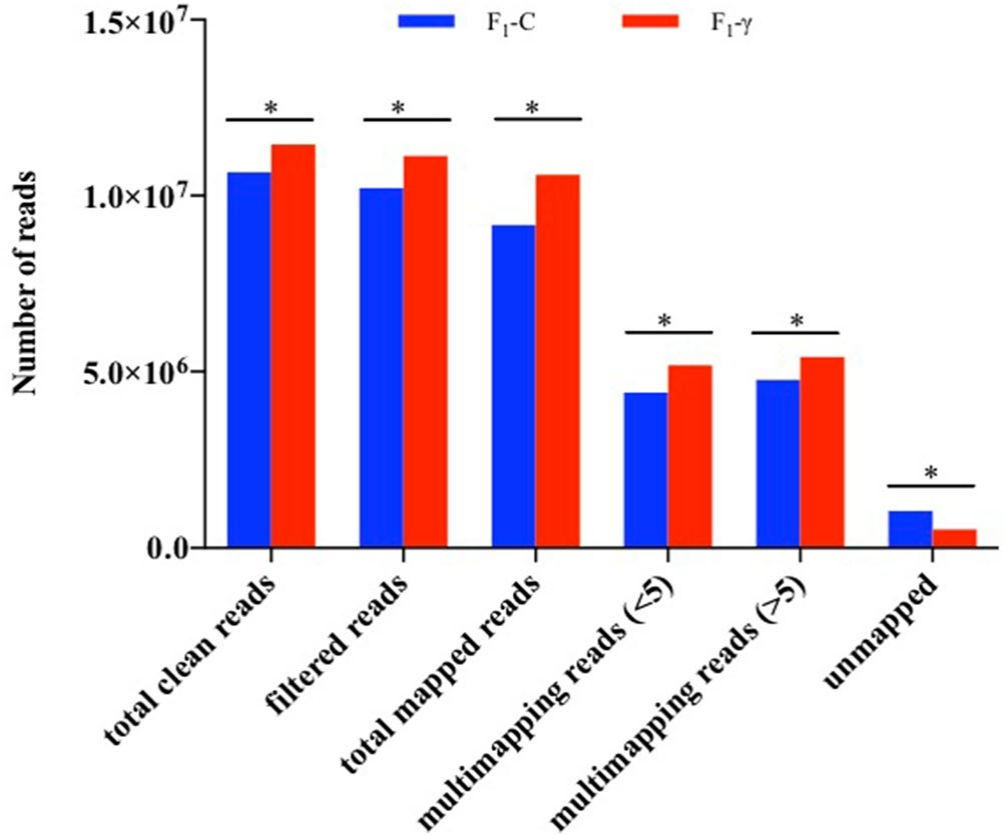
General statistics of reads mapping to zebrafish genome (GRCz10). Three biological replicate libraries per group of F_1_ generation (5.5 hpf embryos) from parents exposed to 8.7 mGy/h of γ-radiation (F_1_-γ) and control parents (F_1_-C). Total clean reads represent the number of reads after QC analysis and filtering. Filtered reads indicate the number of reads after size filtering. Total mapped reads indicate the number of reads mapped to zebrafish reference genome after size filtering. Multimapping reads < 5 include reads aligning to < 5 genomic locations. Multimapping reads > 5, indicate reads mapped to > 5 genomic locations. Unmapped reads show the amount of reads which could not be aligned to the genome. Asterisks represent significant differences p < 0.0001, Chi-square with Yates correction of continuity.

In average, 11,970,959 and 11,389,554 reads resulted from sequencing of F_1_-γ (8.7 mGy/h) and control samples (F_1_-C), respectively. All sequencing libraries had a Phred score higher than 20; however, sequencing reads were filtered by quality using a Phred score of 30. Approximately 95% of reads were recovered after quality filtering (Phred score > 30) over all libraries (data not shown). The read length distribution showed three main peaks present in all libraries, which corresponds to the theoretical sizes of miRNAs (22 nt), piRNAs (26–31 nt), and tRNA-halves (32–34 nt) (Fig. 1). Mapped reads, showed significant differences between offspring of gamma-exposed parents (F_1_-γ) compared to controls (F_1_-C) (80.4% and 88.5%, respectively, p < 0.0001) (Fig. 2). An average of 47.6% of the mapped reads over all libraries aligned to multiple loci (> 5) on the zebrafish reference genome (Fig. 2). It is well known that reads aligning to features such as miRNAs, piRNAs, and tRNA-derived fragments align to multiple genomic regions, including repetitive elements^11,15,16^.

**Figure 3:**
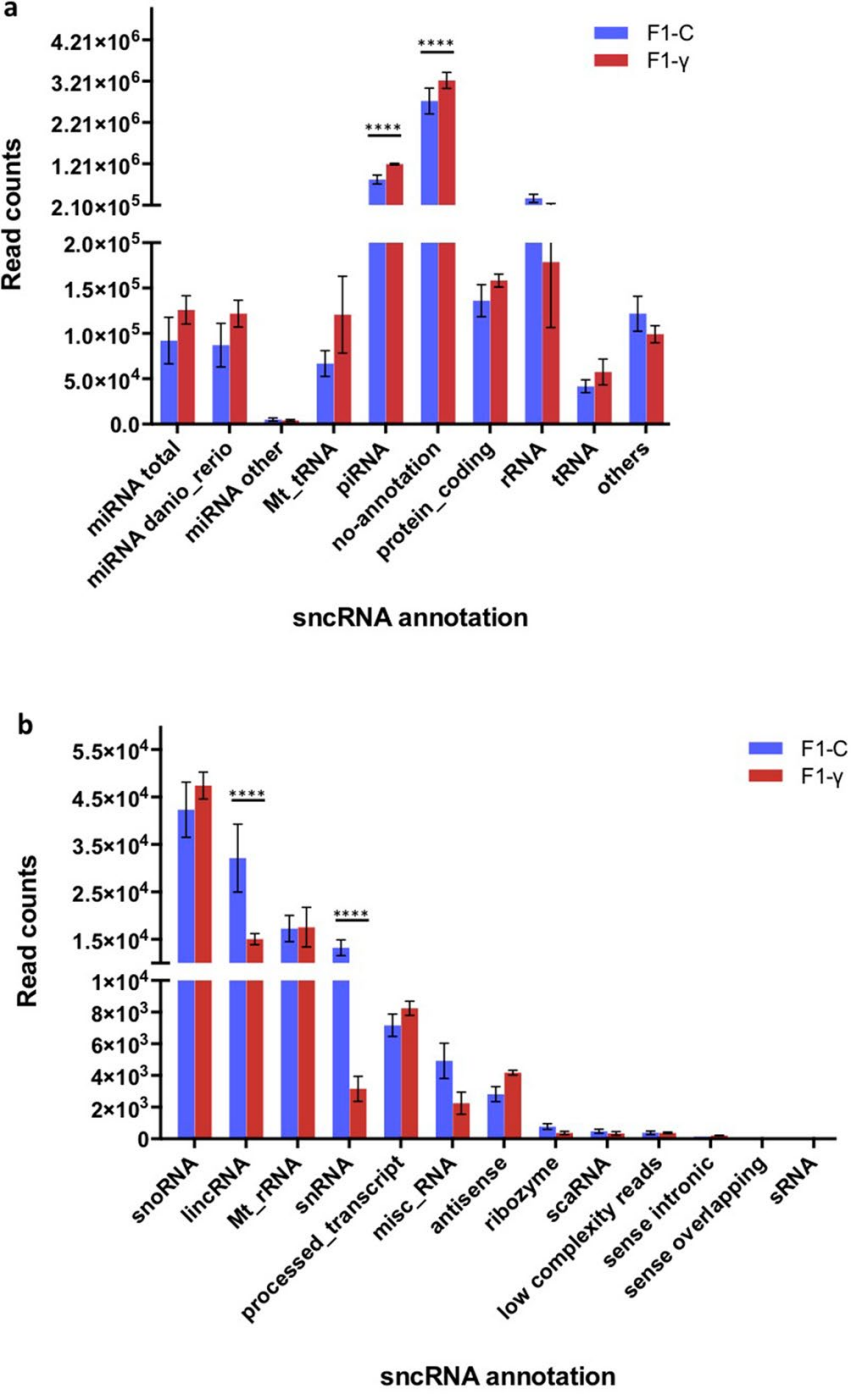
Distribution of mapped reads (zebrafish genome reference GRCz10) onto genomic features. F_1_ generation of embryos (5.5 hpf) from parents 1 year after exposure to 8.7 mGy/h γ-radiation (F_1_-γ) and control parents (F_1_-C). Values derived from three replicates in each group (n = 3). Asterisks denote statistical differences with p < 0.001, multiple t tests (FDR 99%). (**A**) miRNA total (total of reads mapping to microRNAs (miRNA)), miRNA danio_rerio (zebrafish miRNAs), miRNA other (miRNAs from other species), Mt_tRNA (mitochondrial transference RNA), piRNA (piwi-interacting RNA), no-annotation (mapped reads, which could not be assigned to any genomic feature), protein_coding (protein coding transcripts), rRNA (ribosomal RNA), tRNA (genomic transference RNA), others (sum of all reads mapping to genomic features presented in (**B**). (**B**) snoRNA (small nucleolar RNA), lincRNA (long intergenic non-coding RNA), Mt_rRNA (mitochondrial ribosomal RNA), snRNA (small nuclear RNA), misc_RNA (miscellaneous RNA), scaRNA (small cajal-body-spacific RNA), sRNA (bacterial small RNA).

Mapped reads in both groups (F_1_-γ and F_1_-C) were extensively annotated to different genomic features (Fig. 3A,B). Reads mapping to piRNA were significantly enriched in the offspring of gamma-exposed parents (F_1_-γ) (1.4-fold, p < 0.001) as compared to the controls (F_1_-C). Whereas those mapping to lincRNA and snRNA were significantly depleted in the offspring from exposed parents (F_1_-γ) (2.1- and 4.1-folds, respectively p < 0.001). We found no differences with regard to the number of reads mapped to miRNAs or any other genomic feature.

### Differential miRNA expression in F_1_ embryos from exposed parents

Normalization of miRNA expression datasets has been discussed in recent studies, and several methods have given controversial results in miRNA normalization^17–19^. In our case, TMM proved to be suitable to normalize the miRNA expression data, and the exploration after normalization showed a clear effect of gamma radiation on miRNA expression in the offspring F_1_ of exposed parents (F_1_-γ), compared to controls (F_1_-C) (Supplementary Figure S1).

**Figure 4:**
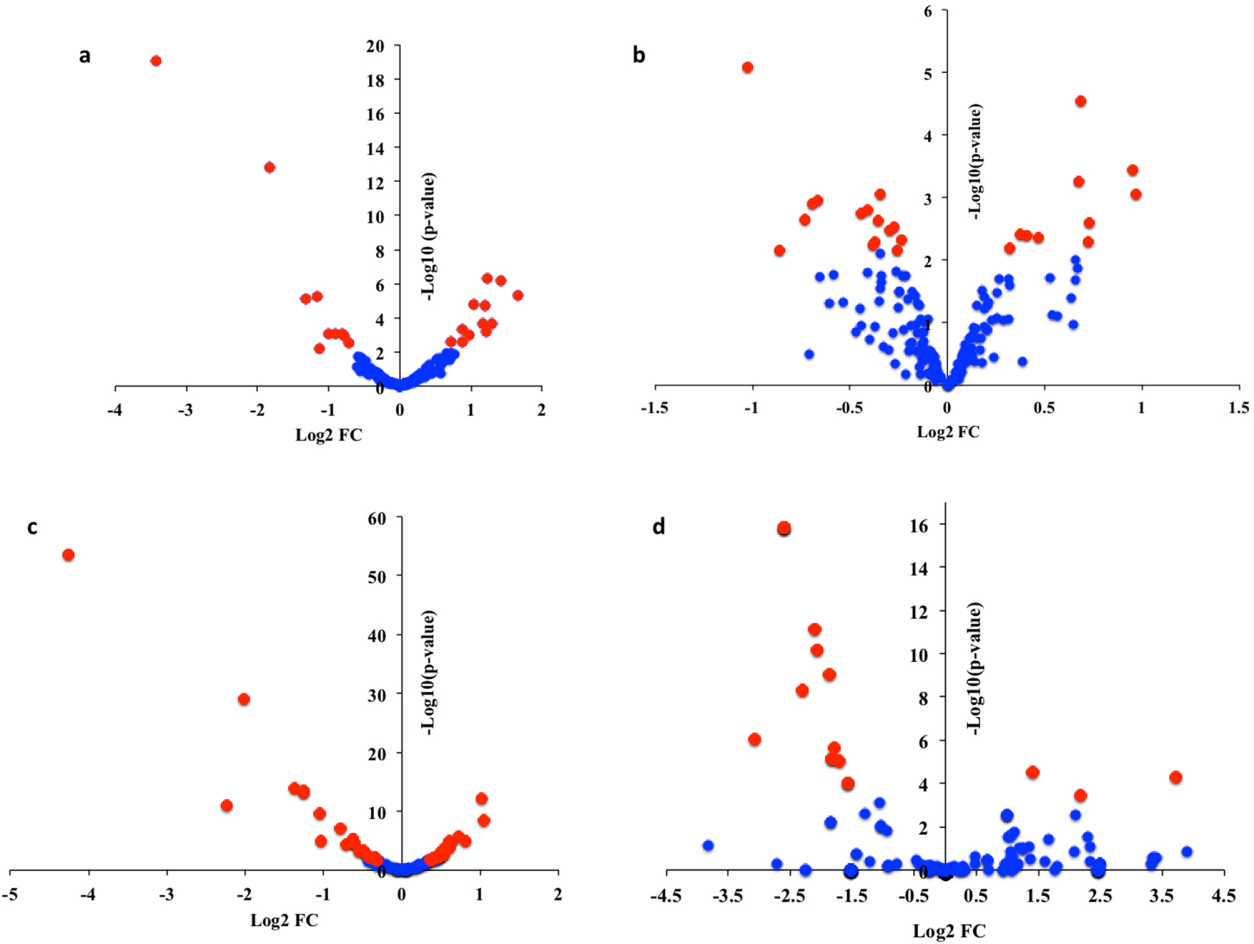
Differential expression analysis of miRNAs (**A**), piRNA clusters (**B**), lincRNAs (**C**), and snRNAs (D) in F_1_ generation of embryos (5.5 hpf) from parents exposed to 8.7 mGy/h γ-radiation (F_1_-γ) and non-exposed control parents (F_1_-C). Differentially expressed genes are represented as red dots. Expression values are shown as log2 of fold changes (X-axis). Y-axis represents the negative log10 of the p values (n = 3).

The differential expression analysis between offspring F_1_ of gamma-exposed parents and controls, using a cut-off of > 30 read counts in all replicates, showed 22 DEmiRNAs (log2 FC > 0.6, p < 0.05, FDR < 0.05), from those, 55% were up-regulated (Fig. 4A; Table 1).

**Table 1:** Differentially expressed miRNAs and their human orthologues.

zebrafish	human	log2FC	FDR	mature sequence ZF	mature sequence human	mismatches	remarks
dre-miR-1-3p	hsa-miR-1-3p	1.65	1.49E-04	u**ggaaugu**aaagaaguauguau	u**ggaaugu**aaagaaguauguau	0	
dre-miR-21-1-3p	hsa-miR-21-5p	1.42	2.49E-05	u**agcuuau**cagacugguguuggc	u**agcuuau**cagacugauguuga	2	
dre-miR-7133-3p		1.29	3.27E-03	ugguguuguguguuaaacugua	N/A		
dre-let-7g-5p	hsa-let-7g-5p	1.22	2.49E-05	u**gagguag**uaguuuguauaguu	u**gagguag**uaguuuguacaguu	1	
dre-miR-150-5p	hsa-miR-150-5p	1.21	8.18E-03	u**cucccaa**uccuuguaccagug	u**cucccaa**cccuuguaccagug	1	
dre-miR-200a-5p	hsa-miR-200a-5p	1.19	3.25E-04	c**aucuuac**cggacagugcugga	c**aucuuac**cggacagugcugga	0	
dre-miR-135c-5p	hsa-miR-135a-5p	1.17	3.19E-03	u**auggcuu**ucuauuccuaugug	u**auggcuu**uuuauuccuauguga	1	
dre-miR-737-5p		1.03	3.25E-04	guuuuuuuagguuuugauuuu	N/A		
dre-miR-204-5p	hsa-miR-204-5p	0.98	8.94E-03	u**ucccuuu**gucauccuaugccu	u**ucccuuu**gucauccuaugccu	0	
dre-miR-458-3p		0.88	1.85E-02	auagcucuuugaaugguacugc	N/A		
dre-miR-30e-3p	hsa-miR-30a-3p	0.88	6.36E-03	c**uuucagu**cggauguuugcagc	c**uuucagu**cggauguuugcagc	0	
dre-let-7j-5p		0.73	1.85E-02	u**gagguag**uuguuuguacaguu	u**gagguag**uagguuguauaguu	3	Not used
dre-miR-27e-3p	hsa-miR-27a-3p	-0.72	2.09E-02	u**ucacagu**ggcuaaguucagug	u**ucacagu**ggcuaaguuccgc	2	
dre-miR-462-5p		-0.78	1.08E-02	uaacggaacccauaaugcagcu	N/A		
dre-miR-141-3p	hsa-miR-141-3p	-0.81	8.69E-03	u**aacacug**ucugguaacgaugc	u**aacacug**ucugguaaagaugg	2	
dre-miR-375-3p	hsa-miR-375-3p	-0.91	8.69E-03	u**uuguucg**uucggcucgcguua	u**uuguucg**uucggcucgcguga	1	Not in IPA
dre-miR-716-3p*		-1.00	8.69E-03	aacgagagctttgaaggcc			Desvignes et al*.,* 2014
dre-miR-181b-5p	hsa-miR-181b-5p	-1.13	4.53E-02	a**acauuca**uugcugucgguggg	a**acauuca**uugcugucggugggu	0	
dre-miR-221-3p		-1.16	1.49E-04	agcuacauugucugcuggguuuc	agcuacauugucugcuggguuuc	0	
dre-miR-2189-3p		-1.32	1.71E-04	ugauuguuuguaucagcugugu	N/A		
dre-miR-205-5p	hsa-miR-205-5p	-1.83	1.26E-11	u**ccuucau**uccaccggagucug	u**ccuucau**uccaccggagucug	0	
dre-miR-738-5p		-3.42	1.44E-17	gcuacggcccgcgucgggaccuc	N/A		

Pairwise comparison between generations F_1_ from exposed (8.7 mGy/h γ-radiation) (F_1_-γ) and control parents (F_1_-C) (log2FC > 0.6, p < 0.05, FDR < 0.05). In bold are the seed sequences. [*] Novel miRNA reported by Desvignes et al.^60^.

Several of the DEmiRNAs listed in Table 1, such as miR-21, let-7g, and miR-150 have previously been reported as affected by ionizing radiation in directly exposed organisms including zebrafish embryos^4,20^. miR-21 is known to act as an oncomiR involved in cancer-related processes^21^, and overexpression of let-7g increase radiosensitivity in lung cancer^4^. In other experimental studies, miR-193b-3p, miR-23b, and members of let-7 family were affected following irradiation^4,21,22^. Besides, DEmiRNAs dre-miR-200a-5p and dre-miR-141-3p, members of family miR-8, are implicated in the control of pluripotency, cancer proliferation, and metastasis^23^. Interestingly, these two DEmiRNAs are oppositely modulated in the offspring of the gamma-exposed parents (Table 1), indicating different regulatory functions. In developmental studies in mouse and zebrafish, this family has been found co-expressed in epithelial and olfactory cells, respectively^24^. In summary, a large fraction of the DEmiRNAs found in offspring F_1_ from gamma-expose parents is related to DEmiRNAs previously found in directly exposed models, indicating that alterations in miRNA expression might be inherited intergenerationally.

### Ingenuity pathway analysis and miRNA target filter

**Figure 5:**
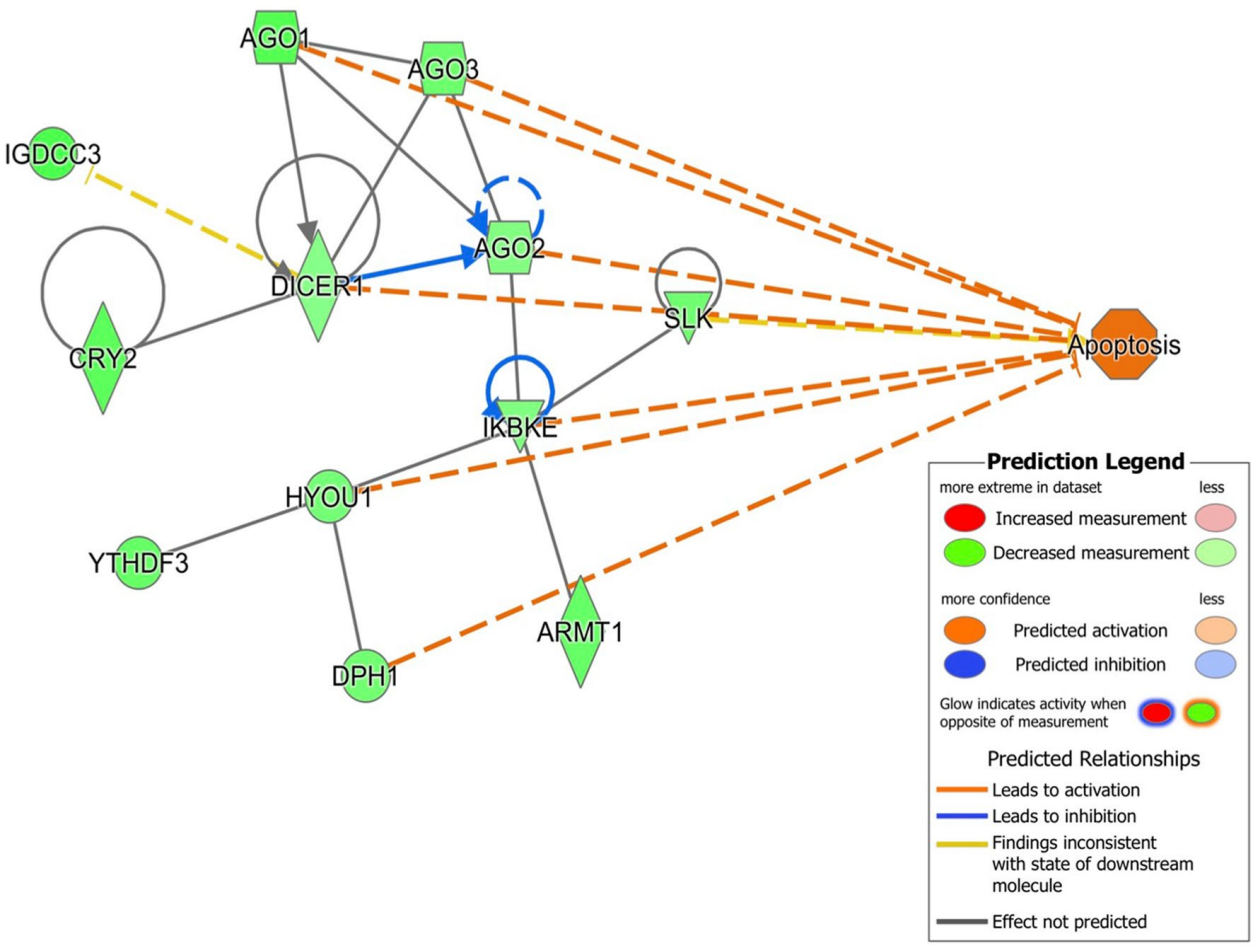
mRNA targets of let-7g involved in apoptosis network and showing the involvement of members of the miRNA biosynthesis pathway as well as other genes. This network predicts activation of apoptosis as shown by the orange edges between nodes. Arrowed blue lines predict inhibition. Green nodes indicate down-regulated genes.

To further explore the possible pathways under miRNA control we used our previously published mRNA dataset (GSE98539)^9^, generated from the same batch of embryos as used in this work, and used the IPA miRNA target filter to link differentially expressed genes (DEGs) to DEmiRNAs (Table 1). This resulted in 12 DEmiRNAs that were imported in IPA of which 11 were in the IPA knowledge base. We found 672 DEGs linked to the DEmiRNAs of which 380 showed an inverse relationship in expression rate with their counteracting DEmiRNA. The pathway analysis on these targets showed overrepresented pathways such as insulin receptor, NFkB, and PTEN signaling (Table 2, Supplementary Data S1). let-7 and miR-21 showed the largest involvement in these specific pathways. Interestingly, IPA’s molecule activity predictor revealed that the involved miRNAs followed the inverse expression relationship as specified in the miRNA target filter (Supplementary Data S1) resulting in predicted effects on apoptosis, transcription and inflammation (Supplementary Figures S2–S4). In directly exposed organisms, including zebrafish embryos, miR-125b acts as a negative regulator of P53, affecting apoptosis^25,26^. In this study, we did not find any modulation of miR-125b in the offspring from gamma-exposed parents (F_1_-γ). However, apoptosis was predicted as an outcome of the interaction of let-7g with a specific network of genes, which included *dicer*, *ago1*, *ago2*, and *ago3* involved in the miRNA biosynthesis pathway (Fig. 5). Furthermore, most overrepresented disease functions were linked to cancer, gastrointestinal and hepatic disease and developmental disorders (Table 2) supporting the observed genomic instability found in larvae from exposed parents by our group^27^ and indicating a vast involvement of miRNAs in DNA damage related response.

**Table 2:** Top significant pathways and disorders (FDR < 0.05) for target genes of differentially expressed miRNAs.

	Pathway	p value	Overlap
Pathways	Insulin receptor signaling	5.39E−05	7.5% (11/147)
	NF-B signaling	1.10E−04	6.4% (12/187)
	PTEN signaling	3.40E−04	7.2% (9/125)
	3-Phosphoinositide Degradation	4.55E−04	6.3% (10/158)
	Huntington's disease signaling	1.50E−03	4.8% (12/250)
Diseases and disorders	Cancer	8.22E−03 to 6.87E−16	358
	Organismal injury and abnormalities	8.23E−03 to 6.87E−16	359
	Gastrointestinal disease	6.73E−03 to 5.12E−12	337
	Hepatic system disease	2.46E−03 to 1.16E−06	249
	Developmental disorder	8.,30E−03 to 1.10E−05	74

### Differential expression of piRNA clusters in F_1_ embryos from exposed parents

In order to determine the expression of piRNAs, candidate piRNAs from both experimental groups (F_1_-γ and F_1_-C) were assigned into clusters. In total, 171 piRNA clusters were predicted from both groups overlapping in genomic position (matched clusters). In concordance with previous studies^11,28^, matched clusters were found expressed from the sense strand (25.7%), antisense strand (48.5%), and bidirectional orientation (25.7%), ranging from 4.3 to 64.0 kb (Supplementary Data S2).

After dataset normalization (Supplementary Figure S5), 11 piRNAs clusters were differentially expressed between offspring of gamma-exposed parents and controls, 6 up-regulated, and 5 down-regulated (log2 FC > 0.6, p < 0.05, Adjusted p value < 0.05; Fig. 4B, Table 3).

**Table 3:** Differentially expressed piRNA clusters.

Clusters	Log2FC	AbsFC	AveExpr	t	P value	Adj.P.Val
Cluster 159c-170g	− 1.02	− 2.03	10.41	− 13.08	8.26E−06	1.41E−03
Cluster 275c-287g	0.68	1.60	10.12	10.69	2.81E−05	2.41E−03
Cluster 160c-171g	0.95	1.93	8.25	6.90	3.66E−04	2.09E−02
Cluster 220c-235g	0.67	1.59	8.67	6.40	5.56E−04	2.38E−02
Cluster 226c-241g	0.96	1.95	8.99	5.91	8.65E−04	2.54E−02
Cluster 230c-244g	− 0.66	− 1.58	8.72	− 5.65	1.10E−03	2.64E−02
Cluster 303c-313g	− 0.69	− 1.61	8.53	− 5.53	1.23E−03	2.64E−02
Cluster 121c-126g	− 0.73	− 1.66	8.28	− 4.96	2.20E−03	3.29E−02
Cluster 63c-60g	0.73	1.65	9.04	4.83	2.51E−03	3.30E−02
Cluster 240c-253g	0.72	1.64	9.54	4.18	5.17E−03	4.21E−02
Cluster 13c-12g	− 0.86	− 1.81	8.83	− 3.91	7.07E−03	4.84E−02

Pairwise comparison between generation F_1_ from exposed (8.7 mGy/h γ-radiation) (F_1_-γ) and control parents (F_1_-C).

**Figure 6:**
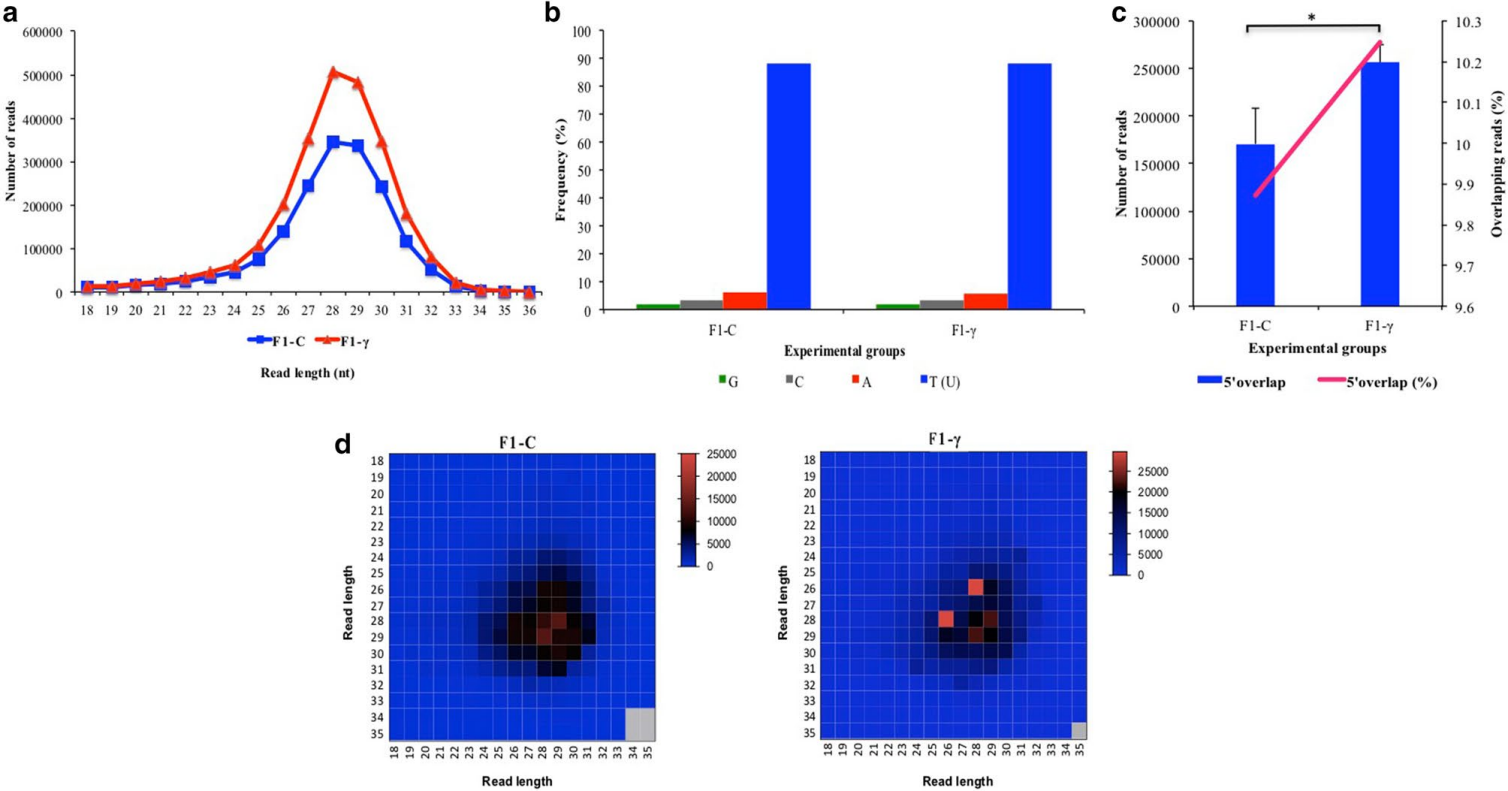
Analysis of piRNA signatures in clusters predicted in F_1_ generation of embryos (5.5 hpf) from parents exposed to 8.7 mGy/h γ-radiation (F_1_-γ) and non-exposed control parents (F_1_-C) (n = 3). A) Read length distribution. B) Per base distribution analysis. C) 5′10nt overlap distribution. Asterisk indicates significant differences (p < 0.05; paired t test). D) Read length analysis of reads with 5′ 10nt overlapping (ping-pong signatures; average Z score F_1_-C = 44.2, and F_1_-γ = 42.9; p < 0.01).

piRNA population within predicted clusters was also searched for piRNA signatures such as uridine (U) at 5′ end, which is recognized as a hallmark of the primary biogenesis pathway, and 10 nt 5′ overlap bias, accepted as a signature for the secondary piRNAs biogenesis pathway, known as ping-pong mechanism^29,30^. Besides the read length (Fig. 6A), found in concordance with the described theoretical size of piRNAs, more than 80% of piRNA reads in both experimental groups contained a U at their 5′end (Fig. 6B). In addition, 9.87% and 10.25% of piRNA candidate reads in F_1_ offspring from exposed and controls parents respectively, showed the typical 10 nt 5′overlap (Fig. 6C). The significantly larger number of reads in the offspring from the gamma-exposed group (F_1_-γ), showing a 10 nt 5′ overlap compared to controls (p < 0.05), suggested a higher abundance of piRNAs in the group F_1_-γ derived from processed transposons through ping-pong mechanism. In zebrafish, Piwil1 process the activated transposons producing secondary piRNAs, which in turn are loaded into Piwil2, generating more piRNAs from primary piRNA transcripts. Thus driving the amplification loop (ping-pong mechanism) resulting in a population a piRNAs of opposite polarity with a 10 nt 5′overlap^31^.

The analysis of ping-pong signatures showed the presence of a similar heterotypic 28 × 29nt peak in both F_1_-γ and control group (F_1_-C). Whereas a strong peak at 26 × 28nt was only found in the offspring form exposed parents (F_1_-γ) (average Z-score F_1_-C = 44.2, and F_1_-γ = 42.9; p < 0.01), indicating that piRNA population in the descendants from the gamma-irradiated parents (F_1_-γ) differs in size from that found in the offspring from the non-irradiated parents (F_1_-C) (Fig. 6D). Since there are only two *piwi* paralogs reported in zebrafish, *piwil1* (piwi-like RNA-mediated gene silencing 1, ENSDARG00000041699) and *piwil2* (piwi-like RNA-mediated gene silencing 2, ENSDARG00000062601), the same peak from 10 nt 5′ overlapping reads might be observed in both experimental groups. Besides, considering that, *piwil2* was found down-regulated when analysing mRNA expression in a parallel study using siblings embryos as for those used in this study^9^, we then hypothesized that parental gamma radiation induced the activation of additional *piwil2* paralog or isoform*,* which might be the cause for the presence of a different peak from 10 nt 5′ overlapping reads in the group F_1_-γ. There are four different *piwil2* isoforms annotated in Ensembl (ENSDART00000090695.7, ENSDART00000162071.2, ENSDART00000134274.3, ENSDART00000136004.2). Nevertheless further research is necessary to prove this hypothesis.

### Differentially expressed piRNA clusters are associated to transposable elements

Although piRNAs appear to be involved in transcriptional regulation and deadenylation of mRNAs^32^, its major role in the gonads is in controlling the expression of TEs, thus protecting the genome from their harmful effects, and ensuring that genetic information is correctly passed down to the next generation^33,34^. Primary piRNA clusters are derived from genomic loci known for harboring TEs. Transcriptionally active TEs are processed by Piwil1 and Piwil2 producing secondary piRNAs (ping-pong mechanism), which are antisense to expressed TEs^31,33^.

We intersected our set of piRNA clusters expressed in the offspring from the gamma-exposed group (F_1_-γ) and the controls (F_1_-C) (matched piRNA clusters) with TEs. As expected, we found a large number of matched piRNA clusters (85 out of 171) overlapping with 172 TEs (p < 0.0001), which were classified into six different orders; LTR, LINE, TIR, Crypton, Helintron and DNA/unknown (Supplementary Data S3). Afterward, we determined a significant association between DEpiRNA clusters and TEs (p = 0.0024), where 45% of DEpiRNA clusters overlapped with 9 TEs belonging to orders LTR, LINE, and TIR. In every case, DEpiRNA clusters appeared to be expressed from the complementary strand of the associated TEs (Table 4), suggesting that these DEpiRNAs have been expressed in response to the activation of TEs.

**Table 4:** Genomic association between DEpiRNA clusters and transposable elements (TE) in F_1_ offspring from parents exposed to gamma radiation.

Class	Order	Superfamily	Transposable element	Chr	Strand	Start	End	Matched clusters	Strand	Coordinate Ctrl	Coordinate gamma
Class I	LTR	ERV	ERV1-N2-LTR_DR	chr4	+	29,601,493	29,601,990	Cluster 159c-170g	−	29,601,046–29,605,599	29,598,133–29,605,596
		Pao	BEL5-LTR_DR	chr5	+	509,591	509,940	Cluster 226c-241g	−	505,497–514,982	503,412–515,021
		Gypsy	Gypsy86-I_DR	chr11	−	15,191,699	15,192,548	Cluster 13c-12g	+	15,189,937–15,194,076	15,189,146–15,194,836
	LINE	L2	L2-41_DRe	chr5	+	510,697	511,321	Cluster 226c-241g	−	505,497–514,982	503,412–515,021
Class II	TIR	hAT	HAT1_DR	chr4	+	72,262,741	72,263,561	Cluster 220c-235g	−	72,259,035–72,270,029	72,259,614–72,270,029
			HAT1_DR	chr4	+	72,265,269	72,265,422	Cluster 220c-235g	−	72,259,035–72,270,029	72,259,614–72,270,029
			HAT1_DR	chr4	+	72,267,726	72,268,391	Cluster 220c-235g	−	72,259,035–72,270,029	72,259,614–72,270,029
		Dada	Dada-tA_DR	chr21	+	30,147,477	30,148,228	Cluster 121c-126g	−	30,146,527–30,153,953	30,145,014–30,153,912
			Dada-tA_DR	chr21	+	30,149,218	30,151,489	Cluster 121c-126g	−	30,146,527–30,153,953	30,145,014–30,153,912

Fisher’s exact test, p-value = 0.0024.

In bovine embryos, piRNAs and TEs of the families LINE and ERV1 among others, that seemed to be associated considering their mapping locations, showed an inverse relationship in expression^35^. Additionally, TEs activation due to irradiation has been observed in yeast, plants, and *Drosophila*^36–38^. In concordance, TEs have been associated with DNA integrity disruption and double-stranded breaks in *Drosophila*^39^. Therefore, parallel experiments on the siblings of these embryos showed a significantly higher DNA damage rate in the offspring of the gamma exposed parents as compared to embryos from control (non-irradiated) parents 1 year after parental irradiation, which lacked of association with an effect from reactive oxygen species^27^.

Though piRNAs-mediated TEs silencing through recruitment of DNA methyltransferase has been proposed as a mechanism for controlling TEs activation in mammals^40^, we found no association between piRNA clusters and DMRs obtained from our parallel study^8^. To our best knowledge, this is the first study reporting an association between altered expression of piRNA clusters and TEs in the generation F_1_ of parents exposed to gamma radiation.

### Differential expression of snRNA in F_1_ embryos from exposed parents

We observed a significantly lower amount of reads mapping to snRNAs in F_1_ offspring from gamma-irradiated parents (F_1_-γ) as compared to the F_1_ offspring of non-irradiated parents (F_1_-C) (4.1-folds, p < 0.001) (Fig. 3B). Using a cutoff of more than 100 read counts, the expression of 25 snRNA genes was detected (Supplementary Data S4). Pairwise comparison on the normalized dataset (Supplementary Figure S6) showed 19 (76%) snRNA genes differentially expressed (DEsnRNA) (log2FC > 0.6, p < 0.05, FDR < 0.05). From those, 16 were down-regulated in the group F_1_-γ, whereas only three snRNAs were up-regulated (Fig. 4D; Table 5).

**Table 5:** Differentially expressed snRNAs.

Feature	ID	Log2FC	AbsFC	logCPM	p-value	FDR
U1.87-201	ENSDART00000122651	− 2.59	− 6.03	16.43	1.45E−16	4.81E−14
U1.78-201	ENSDART00000130858	− 2.59	− 6.03	16.43	1.47E−16	4.81E−14
U1.63-201	ENSDART00000131127	− 2.59	− 6.03	16.43	1.51E−16	4.81E−14
U1.30-201	ENSDART00000118247	− 2.59	− 6.03	16.43	1.56E−16	4.81E−14
U2.11-201	ENSDART00000115842	− 2.10	− 4.31	14.75	7.34E−12	1.80E−09
U2.4-201	ENSDART00000118922	− 2.07	− 4.20	14.86	6.71E−11	1.37E−08
U2.10-201	ENSDART00000116995	− 1.86	− 3.63	14.96	9.03E−10	1.59E−07
U4.29-201	ENSDART00000118843	− 2.29	− 4.92	13.74	4.70E−09	7.23E−07
U5.9-201	ENSDART00000120566	− 3.07	− 8.44	11.77	9.00E−07	1.23E−04
U12.1-201	ENSDART00000115597	− 1.80	− 3.48	12.27	2.36E−06	2.90E−04
U1.34-201	ENSDART00000118029	− 1.83	− 3.57	12.43	7.50E−06	7.69E−04
U1.46-201	ENSDART00000126252	− 1.83	− 3.57	12.43	7.51E−06	7.69E−04
U5.5-201	ENSDART00000115886	− 1.72	− 3.29	12.75	9.18E−06	8.67E−04
U1.229-201	ENSDART00000165185	1.40	2.64	11.64	2.85E−05	2.50E−03
U1.194-201	ENSDART00000169715	3.71	13.13	8.59	4.90E−05	4.01E−03
U1.74-201	ENSDART00000128520	− 1.57	− 2.96	12.51	1.01E−04	7.29E−03
U1.16-201	ENSDART00000118626	− 1.57	− 2.96	12.51	1.01E−04	7.29E−03
U1.221-201	ENSDART00000161457	2.16	4.49	10.99	3.40E−04	2.32E−02
U4.25-201	ENSDART00000115598	− 1.05	− 2.07	11.82	7.58E−04	4.90E−02

Pairwise comparison between generations F_1_ from exposed (8.7 mGy/h γ-radiation) (F_1_-γ) and control parents (F_1_-C). Transcript IDs were obtained from Ensembl database (http://www.ensembl.org).

Small nuclear RNAs comprise a group of nuclear-localized sncRNAs, which are critical components of the spliceosome. From the five major spliceosomal snRNA (U1, U2, U4, U5, and U6), the snRNA U1 and U2 guide the association of the spliceosome to the 5′ and 3′splice sites respectively, and are essential for the early splicing process^41^. Interestingly, the vast majority of down-regulated snRNA genes (12 out of 16) in the group F_1_-γ were members of U1 and U2. In addition to U1 and U2, four members of U4 and U5 were found down-regulated. In total, 4 out of 5 major spliceosomal snRNAs were represented in our data as down-regulated genes (Table 5).

We hypothesize that the down-regulation of the major spliceosomal snRNAs negatively affects the gene expression rate. Both snRNAs U1 and U2 participate in the regulation of transcription by increasing the formation of the first phosphodiester bond during transcription initiation, and also interact with components of the initiation complex such as TFIIH (transcription factor II human)^41–43^. On the other hand, *gtf2h1* (general transcription factor 2 h polypeptide 1), a gene encoding for a subunit of TfIIh, was found down-regulated when analysing mRNA expression of siblings embryos as for those used in this study^9^. The later suggests that combined down-regulation of the major spliceosomal snRNAs and *gtf2h1* can be the cause of the lower global gene expression rate in F_1_ offspring of gamma irradiated parents from our parallel gene expression study^9^.

### Differential expression of lincRNA in F_1_ embryos from exposed parents

We found a significantly lower amount of reads mapping to lincRNA genes in the embryos from parents exposed to gamma radiation (F_1_-γ) than in controls (F_1_-C) (2.1-folds, p < 0.001) (Fig. 3B).

Aiming to compare the expression of lincRNAs between F_1_-γ and control (F_1_-C) embryos, the reads mapping to lincRNAs in both groups were quantitated. We detected the expression of 108 lincRNA genes with a cut-off of more than 100 read counts (Supplementary Data S5). After normalization of the quantitated dataset (Supplementary Figure S7), 44 lincRNAs were differentially expressed (DElincRNA) in the group F_1_-γ (Fig. 4C). We shortlisted the number of DElincRNAs to 21 genes using a more stringent fold change cut-off (log2FC > 0.6, p < 0.05, FDR < 0.05) that could be categorized into 14 (66.7%) down-regulated and 7 (33.3%) up-regulated lincRNAs in the offspring of the gamma irradiated parents (F_1_-γ) (Table 6).

**Table 6:** Differentially expressed lincRNAs.

Transcript ID	Name	Log2FC	AbsFC	logCPM	P value	FDR
ENSDART00000151198.3	si:dkey-153m14.1	− 4.26	− 19.20	16.75	2.85E−54	3.08E−52
ENSDART00000163316.2	malat1-001	− 2.01	− 4.03	10.91	8.88E−30	4.79E−28
ENSDART00000152334.2	si:ch211-202n12.4-001	− 1.37	− 2.58	11.34	1.08E−14	3.88E−13
ENSDART00000142034.2	si:dkey-163i6.7-001	− 1.25	− 2.37	10.42	3.66E−14	9.89E−13
ENSDART00000153803.3	si:ch73-112l6.6-001	− 1.26	− 2.39	10.41	6.64E−14	1.44E−12
ENSDART00000155205.2	si:ch73-65n21.2-001	1.01	2.02	13.76	6.85E−13	1.23E−11
ENSDART00000171481.2	si:dkey-23f9.15-001	− 2.23	− 4.69	12.80	1.01E−11	1.56E−10
ENSDART00000157228.2	si:dkeyp-9a1.3-001	− 1.05	− 2.07	13.97	2.17E−10	2.93E−09
ENSDART00000155833.2	si:dkey-30f11.2-001	1.04	2.06	12.71	4.11E−09	4.93E−08
ENSDART00000161654.2	si:dkey-208k4.7-002	− 0.79	− 1.72	12.14	9.07E−08	9.80E−07
ENSDART00000194508.1	N/A	0.72	1.65	12.64	2.20E−06	2.16E−05
ENSDART00000164487.2	si:zfos-754c12.1-001	− 0.61	− 1.53	14.54	5.31E−06	4.78E−05
ENSDART00000157923.2	si:ch211-209n20.60-001	0.80	1.75	12.57	1.14E−05	8.53E−05
ENSDART00000145442.3	si:ch211-209a2.1-001	− 1.03	− 2.04	11.95	1.18E−05	8.53E−05
ENSDART00000184251.1	N/A	0.61	1.52	13.29	1.18E−05	8.53E−05
ENSDART00000155805.2	si:dkey-273g18.4-001	0.63	1.55	11.89	1.51E−05	1.02E−04
ENSDART00000173530.2	N/A	− 0.63	− 1.54	12.03	2.01E−05	1.28E−04
ENSDART00000135637.2	si:dkeyp-116h7.2-001	− 0.60	− 1.52	13.19	2.43E−05	1.46E−04
ENSDART00000154612.2	si:dkeyp-116h7.4-001	− 0.60	− 1.52	13.19	2.82E−05	1.60E−04
ENSDART00000155864.3	si:zfos-1451h6.1-001	− 0.71	− 1.64	12.91	5.31E−05	2.87E−04
ENSDART00000164620.2	si:dkey-250k10.3-001	0.59	1.51	12.59	1.82E−04	8.93E−04

Pairwise comparison between generations F_1_ from exposed (8.7 mGy/h γ-radiation) (F_1_-γ) and control parents (F_1_-C). Transcript IDs and transcript names obtained from Ensembl database (http://www.ensembl.org) and ZFIN (The Zebrafish Information Network; http://zfin.org/) respectively.

To gain insight into the biological function of the DElincRNAs, we searched for DElincRNAs within our dataset with conservation and functional annotation in the ZFLNCRNA database^44^. Only five out of 21 DElincRNAs were found conserved in human or mouse representing the 23.8% (Table 7). Similarly, previous studies found only 5.3 to 8.9% of zebrafish lncRNAs conserved^45,46^.

**Table 7:** List of differentially expressed lincRNAs in the generation F_1_ from exposed (8.7 mGy/h γ-radiation) (F_1_-γ) parents with conserved orthologs in human and/or mouse and functional annotations.

LincRNA	ZFLNCRNAdb	Chr	Start	End	Strand	Human	Mouse	GO	KEGG
ENSDART00000157923.2	ZFLNCT04065	chr4	38,126,500	38,135,543	−	No	NONMMUT042657	No	No
ENSDART00000163316.2	ZFLNCT12715	chr14	46,643,943	46,651,420	−	ENST00000534336	ENSMUST00000172812	Yes	Yes
ENSDART00000151198.3	N/A	chr20	55,338,635	55,339,525	−	lncAB371.6	No	N/A	N/A
ENSDART00000142034.2	ZFLNCT17985	chr21	22,330,675	22,338,370	−	No	ENSMUST00000144118	Yes	Yes
ENSDART00000153803.3	ZFLNCT17986	chr21	22,333,232	22,335,576	+	No	ENSMUST00000144118	Yes	Yes
ENSDART00000145442.3	ZFLNCT05491	chr5	60,665,272	60,669,808	+	No	No	Yes	Yes
ENSDART00000155864.3	ZFLNCT10158	chr11	7,139,664	7,141,407	+	No	No	Yes	Yes
ENSDART00000135637.2	ZFLNCT17908	chr21	17,947,814	17,976,724	−	No	No	Yes	Yes
ENSDART00000154612.2	ZFLNCT17909	chr21	17,948,235	17,957,900	+	No	No	Yes	Yes
ENSDART00000164487.2	ZFLNCT10938	chr12	20,371,009	20,378,455	+	No	No	Yes	Yes

Transcript IDs obtained from Ensembl database (http://www.ensembl.org) and ZFLNCRNA Database (http://www.zflnc.org) respectively.

Conserved genes are thought to exert similar biological functions across species. One of the most down-regulated DElincRNAs found in the descendants from gamma-irradiated parents represents an ortholog of the well-characterised human lincRNA MALAT1 (metastasis associated lung adenocarcinoma transcript 1) (Tables 6, 7). MALAT1 has been shown to interact with several serine/arginine (SR) proteins, driving the distribution of splicing factors in the nucleus. The down-regulation of MALAT1 in HeLa cells resulted in decreased association of splicing factors to the nuclear speckle, producing alterations in the alternative splicing of endogenous pre-mRNA^47^. Besides, cellular responses to radiation exposure, DNA damage, and apoptosis can affect the alternative splicing process^48^. Therefore, the strong down-regulation of *malat-1*, and the down-regulation of major spliceosomal snRNAs suggest alterations in the alternative splicing in the generation F_1_ from exposed parents.

In addition, some lincRNAs can target miRNAs and counteract their function by reducing specific miRNA availability^49^. We used the experimental module from DIANA-LncBase (v.2)^50^ to search for experimentally confirmed miRNA-lncRNA interactions. Human orthologs of miRNAs dre-miR-21-1-3p, dre-let-7g-5p, dre-miR-1-3p, and dre-miR-135c-5p, among others (Table 1), were found as targets of MALAT1 (Supplementary Figure S8). This set represent up-regulated miRNAs in our dataset in contrast to the down-regulation of *malat-1*. Our pathway analysis indicated the involvement of those DEmiRNAs in the regulation of pathways such as insulin receptor-signaling, NFKB signaling, and pTEN (Supplementary Figures S2, S3, S4).

On the other hand, in zebrafish embryos, the knockdown of *malat-1* caused a high mortality rate and various types of phenotypical alterations^51^. This is in concordance with the expression profile of *malat-1* in our data, suggesting that its down-regulation contributed to establishing altered phenotypes documented in the F_1_ offspring from gamma-irradiated parents^9,27^. To the best of our knowledge the rest of conserved DElincRNAs represent uncharacterised members of this class, without available biological information.

**Figure 7:**
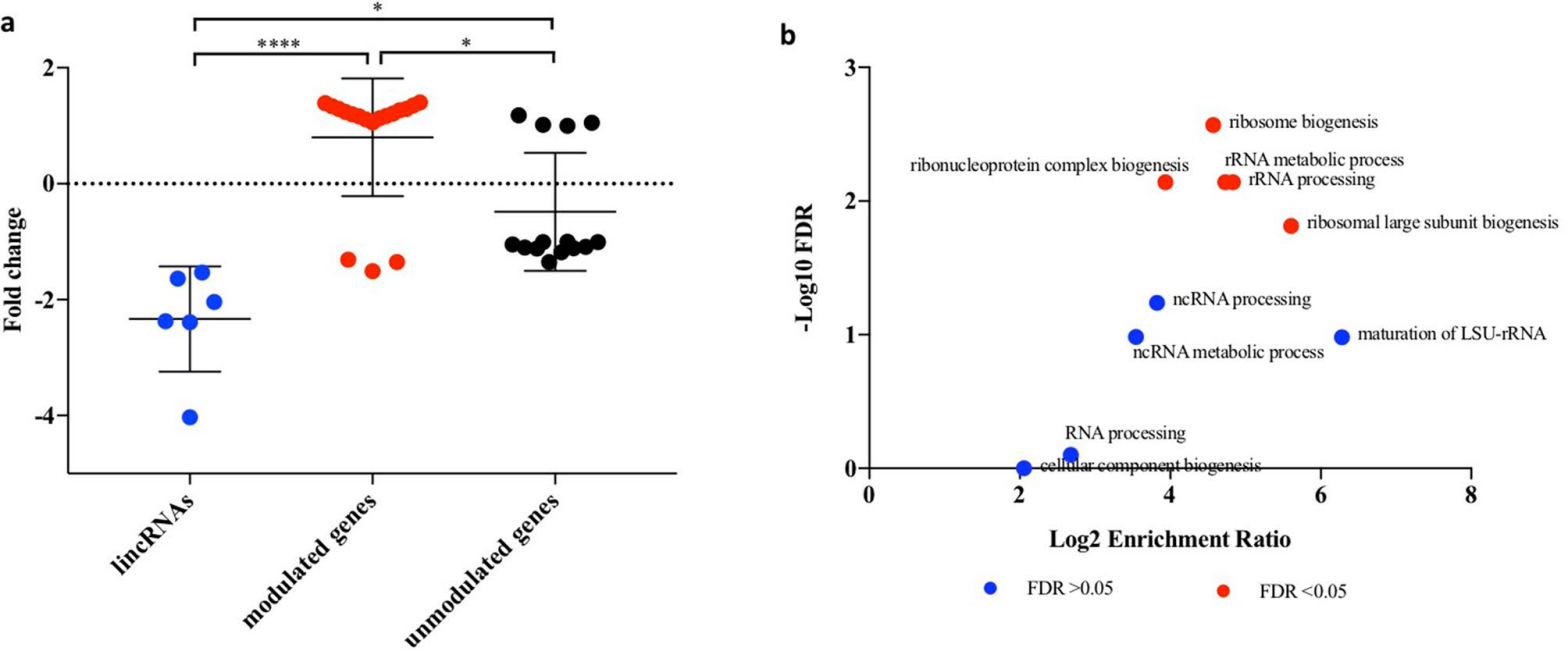
Expression and biological processes of genes within the co-expression network of DElincRNAs in the generation F_1_ of embryos (5.5 hpf) from parents exposed to 8.7 mGy/h γ-radiation (F_1_-γ). Gene expression values from our parallel differential expression data (GSE98539)^9^. (**A**) DElincRNAs expression level compared to modulated and unmodulated genes within the co-expression network. Modulated genes represent differentially expressed (FDR < 0.05), unmodulated genes represent not differentially expressed (FDR > 0.05). Asterisks indicate significant differences (**** p < 0.0001, * p < 0.05, Kruskal–Wallis and Dunn’s multiple comparison test, n = 3). (**B**) Overrepresented GO terms under the category biological processes from modulated genes in A (FDR < 0.05 Benjamini–Hochberg).

We retrieved the co-expression networks of those DElincRNAs in our data with functional annotation in the ZFLNCRNA database (Table 7). This resulted in a subset of 59 genes representing unique entries. When intersecting this subset of genes with our previously published parallel mRNA-Seq data^9^, 32 genes (54.2%) had quantitated expression levels, whereas 27 genes (45.8%) were not found (Supplementary Data S6). The 32 genes detected were classified based on their expression levels as modulated (FDR < 0.05) or unmodulated (FDR > 0.05). The vast majority of modulated genes showed expression levels opposite to the DElincRNAs, while unmodulated genes had significantly lower expression levels as compared to modulated genes (p < 0.05) (Fig. 7A). This indicates that down-regulation of DElincRNAs result in increased expression of genes within the co-expression networks. The GO analysis of modulated genes under the category of biological processes showed significant enrichment in five GO terms (FDR < 0.05), such as ribosome biogenesis (GO:0042254), ribonucleoprotein complex biogenesis (GO:0022613), rRNA metabolic process (GO:0016072), rRNA processing (GO:0006364), and ribosomal large subunit biogenesis (GO:0042273) (Fig. 7B). Besides, the pathway ribosome biogenesis in eukaryotes (ID 03009) was found overrepresented (p value 2.406E−05, FDR 4.019E−03). The GO analysis of unmodulated genes did not show any GO terms significantly overrepresented.

The synthesis of ribosomes must be under strict control to guarantee correct cell growth and proliferation. Disturbances in this pathway result in altered cell cycle and proliferation, similar to that observed in cancer^52^. In addition, the ribosome biogenesis pathway is sensitive to radiation in directly exposed mice^53^.

Based on publicly available data we have analysed the function of a small subset of DElincRNAs. We lack information about to the biological function of many other DElincRNAs in our data, limits our capacity to unravel all the implications of the dysregulation of this ncRNA class, in the descendants of parents exposed to gamma radiation. Despite not being fully understood, our results show the potential involvement of DElincRNAs in the regulation of miRNAs responsive to gamma radiation, post-transcriptional processes, and protein synthesis.

## Conclusions

Our results show the effect of gamma radiation on the non-coding transcriptome in the first-generation offspring of exposed parents. The exposure of adult zebrafish during gametogenesis to a dose rate similar to that observed in Chernobyl 60 days post-accident, produced alterations on the expression profile of different classes of ncRNAs such as miRNAs, piRNAs, snRNAs, and lincRNAs, which were observable 1 year later in the generation F_1_. Moreover, pathway analysis, and ncRNA expression could be linked to previously observed gamma radiation effects on phenotype and gene expression of these embryos. Altogether, our results provide new knowledge on the involvement of ncRNAs in the response to gamma radiation, and contribute to better understand to what extent the adverse effects of gamma radiation are inherited by offspring. Furthermore, this work sets the bases for transgenerational studies, with a focus on mechanisms of inheritance, expression pattern maintenance, as well as the possible impact on population dynamics.

## Material and methods

### Zebrafish husbandry and exposures

Zebrafish strain AB wild type was obtained from the Norwegian University of Life Sciences (NMBU) zebrafish facility and kept according to standard operational procedures as previously described by Hurem et al.^7^. The exposures of fish to gamma radiation, mating, as well as the generation of embryos were carried out as previously described by Hurem et al*.*^27^. Briefly, adult zebrafish (6 months old) were exposed for 27 days to a ^60^Co source at 8.7 mGy/h (5.2 Gy total dose), which represents a similar dose rate to that observed in Chernobyl 60 days post-accident^54^. Control fish were kept separately under similar experimental conditions. In both exposure and control groups, three replicates of 30 males and 30 females were used.

This study was conducted under the approval of the Institutional Animal Ethics Committee (IACUC) and the Norwegian Food Inspection Authority (NFIA), under permit number 5793. NMBU zebrafish facility, is licensed by the NFIA and accredited by the association for assessment and accreditation of laboratory animal care (AAALAC, license number: 2014/225976). The NMBU zebrafish facility and SOPs has AAALAC accreditation (No. 1036) and is approved by the National Animal Research Authority. All experiments were performed according to Norwegian Animal Welfare Act (2009) and the EU Directive 2010/63, following appropriate guidelines.

### Embryo sampling

One year after exposure, fish were mated, and embryos were obtained as previously described by Hurem et al.^27^. First-generation embryos from both, exposed and control parents were harvested in 100 mm Petri dishes (Costar, Corning incorporated, USA) containing autoclaved system water. The embryos were incubated at a controlled temperature of 28 ± 2 °C until staging at 5.5 hpf (50% epiboly). The autoclaved system water was replaced every two hours. The staging of embryos was performed following Kimmel et al.^55^. Staged embryos from each experimental group were directly transferred into 12-well plate (Costar, Corning incorporated, USA), containing 3 mL of autoclaved system water at a controlled temperature of 28 ± 2 °C. Pools of 100 embryos from each replicate in both experimental groups were allocated in separate wells, and washed 3 times with 10 mL autoclaved system water (28 ± 2 °C) to remove debris from the previous incubation period. Afterward, each pool of embryos was transferred to 1.5 mL microfuge tubes (Thermo Fisher Scientific, Waltham, MA) and snap-frozen in liquid nitrogen. All samples were stored at − 80 °C for further analysis.

### RNA isolation

Batches of 100 embryos in triplicates were homogenized using Magnalyser Beads (Roche Diagnostics, Germany), and total RNA was isolated using Trizol following the instructions of the manufacturer (Thermo Fisher Scientific, USA). Exogenous synthetic kanamycin mRNA (Promega, USA) was added as spike-in RNA to the Trizol (0.25 ng/mL). RNA integrity and concentration were assessed with Bioanalyzer using the RNA Nano LabChip Kit (Agilent Technologies, Santa Clara, CA) and Nanodrop 1000 (Thermo Fisher Scientific, USA) respectively. RNA samples with RIN values above eight were stored at − 80 °C until used for sequencing.

### Small RNA sequencing

The total RNA samples were sent for custom sequencing (Novogen, Hong Kong) under Illumina platform (HiSeq 4000). Three single-end libraries (biological replicates) from the exposure group (F_1_-γ), and the control group (F_1_-C) were made following the manufacture’s recommendations. In short, sequencing libraries were prepared from each sample using 1 μg of total RNA as input material (NEBnext Small RNA Library Prep Set for Illumina, New England Biolabs inc, USA). Adaptors at 5′and 3′-ends were ligated to generate non-directional cDNA libraries. After reverse transcription, cDNA was amplified using 12 PCR cycles. Amplified cDNA libraries were purified using AMPpure XP beads kit and size-fractionated using 6% polyacrylamide gel to obtain the fraction corresponding to sncRNAs (up to 150 bp). Libraries were checked for quality using Bioanalyzer 2100 system (Agilent Technologies, Palo Alto, USA) with DNA 1000 chip kit (Agilent Technologies). The libraries, with read lengths of 50 nt, were sequenced with targeted depth 10 million reads per library. Raw reads were deposited at Gene Expression Omnibus Database under accession number GSE152189.

### Bioinformatics analysis

Raw reads were trimmed from adapter sequences and quality assessed using Trim Galore! v0.3.7^56,57^. Only reads with Phred score > 30 were kept for further analysis. After adaptor trimming and QC filtering, raw reads were filtered by length using a combination of on terminal awk, unix, and perl commands. Only reads within the range 18–36 nt were retained. Length filtered reads were then mapped to zebrafish reference genome GRCz10 (http://www.ensembl.org), classified into genomic features, and counted using Unitas (v1.5.2)^30^ under default settings.

We used miRDeep2 v0.0.5^58^ to quantify miRNAs using the built-in script quantifier.pl, the option -W was used to weight read counts by their number of mapped loci. Options -p, -m, -r and -t were used to indicate miRNA precursor reference, mature miRNA reference, read files in FASTA format, and species, respectively. The rest of the options were kept as default. Precursor and mature miRNA references were obtained from miRBase v21 (http://www.mirbase.org)^59^, and updated with other reported miRNAs^60^.

Reads mapping to piRNA producing loci (piRNA cluster database released 01.08.2018, http://www.smallRNAgroup-mainz.de) and mapped reads that were not assigned to any known genomic feature were subsequently used for piRNA analysis. We first determined ping-pong signatures using Unitas (v1.5.2)^30^ under the option -pp, to determine the 5′ 10 bp overlaps among all potentially piRNA mapped reads.

Once ping-pong signatures were determined, we proceeded to group piRNA reads by clusters to predict piRNA producing loci using proTRAC (v4.2.4)^34^. As pre-processes, we used the perl script TBr2-duster.pl (v2.1)^61^ under default values to collapse mapped reads and remove low complexity sequences. Afterward, reads were remapped using sRNAmapper (v1.0.5)^61^ to generate the input files for downstream processes. All options were left to default settings except for -alignment, which was set to best. Due to the presence of multimapping reads, the script reallocate.pl (v1.1, http://www.smallRNAgroup-mainz.de) was used to reallocate multimapping reads based on the calculation of estimated expression rates of uniquely mapping reads. The output files obtained from the reallocation step were used as input for proTRAC (v4.2.4)^34^ under default values. A zebrafish repeat masker annotation for GRCz10 was obtained from http://www.repeatmasker.org and passed onto proTRAC.

Bedtools package (v2.29.1)^62^ with the subcommand intersect was used to intersect the genomic coordinates of predicted piRNA clusters from the three replicates of each experimental group. The option -f was set to 0.50 to report any overlap of piRNA clusters between replicates within each group by more than 50%. Any piRNA cluster not found in all replicates was removed and not considered for further analysis.

Aiming to associate piRNA clusters to transposable elements (TEs), Bedtools package (v2.29.1)^62^ under the same settings mentioned above, was used to intersecting the genomic coordinate of differentially expressed piRNA clusters (DEpiRNAs) in both experimental groups, and the previously downloaded repeat annotation reference. We also used previously published data from our group in an attempt to link piRNA clusters to previously published DNA methylation changes observed in the same batch of embryos used in this study^8^. Therefore, we used the genomic coordinates of the DEpiRNAs and overlapped those with observed differentially methylated regions (DMRs) (GSE100470) using Seqmonk^63^.

### Differential expression analysis and statistics

Statistical differences in global read counts for reads mapped to genomic features between experimental groups were obtained by multiple t-tests followed by False Discovery Rate (FDR) calculation (Benjamini, Krieger, and Yekutieli method, Q = 1%.).

Genomic features such as miRNA, piRNA clusters, snRNA, and lincRNA (long intergenic non-coding RNA), were analysed for differential expression. All differential expression analyses were performed following the same analytical sequence as follows; data exploration, normalization, data exploration post-normalization, and pairwise comparison between experimental groups (F_1_-γ and F_1_-C; n = 3). Pre-normalization data log2 transformed was explored for descriptive statistics such as minimum, first quartile, median, third quartile, and maximum also the similarity among samples was determined by correlation and hclust (Ward method) analysis to determine the distance between samples. Multidimensional Scaling Plot (MDS-plot) was used to analyze the variances, except for piRNA clusters where Principal Component Analysis (PCA) was utilized.

miRNA, lincRNA, and snRNA expression datasets were TMM normalized (trimmed mean of M-values, edgeR v3.24.3, Bioconductor)^64^, whereas piRNA clusters dataset was RPKM normalized to correct for differences in clusters length. Post-normalization data exploration was conducted similarly to pre-normalization, in both cases, the statistical package R v3.0.2^65^ was used.

Normalized datasets were used for differential expression analysis. In the case of miRNAs, lincRNAs, and snRNAs, the statistical analysis was based on the pairwise comparison between treatment (F_1_-γ) and control (F_1_-C) (n = 3). Experimental groups were compared using the exact test under Bioconductor package edgeR v3.24.3^64^. As for piRNA clusters, F_1_-γ and F_1_-C groups were compared through a pairwise comparison using the function voomWithQualityWeights followed by a linear model and eBayes from Bioconductor package limma (v3.36.3)^66^.

In all analyses, the FDR was set up to 95%. Only ncRNAs with significant FDR (p > 0.05) and log2 FC > 0.6 were considered as differentially expressed.

Fischer’s exact test was used to search for overrepresentation by comparing the number of piRNA clusters overlapping all methylated regions and piRNA clusters merely overlapping differentially methylated regions (DMRs), as well as TEs overlapping with all piRNA clusters and those overlapping to DEpiRNA clusters. Kruskal–Wallis test followed by Dunn’s multiple comparison (alpha 0.05, confidence level 95%) was used to compare the expression of lincRNAs, to genes classified as modulated, and unmodulated within the lincRNA co-expression networks.

### Functional analyses

In order to use the miRNA target filter in Ingenuity Pathway Analysis (IPA, Qiagen, USA), the DEmiRNAs were manually converted to their human orthologue via miRBase^59^. Only miRNAs with a 100% match in seed sequence and a maximum of 2 mismatches in the mature sequence were used in the downstream analysis (Table 1). Within IPA, the miRNA list was linked to our previously published differentially expressed mRNAs (DEGs) (GSE98539)^9^. The miRNA target filter is based on computational as well as experimental evidence of miRNA targets. After filtering the target list to canonical targets (miRNA up-regulated vs target mRNA down-regulated, or vice versa), pathway analysis was performed.

FASTA sequences of DElincRNAs (Supplementary Data S7) were obtained from UCSC Genome Browser (http://genome.ucsc.edu) using the tool Table Browser. The downloaded FASTA sequences were then annotated by BLAST against the Zebrafish LncRNA Database (ZFLNCRNA, http://www.zflnc.org)^44^ under default parameters (e-value 0.001, word size 11, sensitivity normal) to search for conservation and functional annotations. DElincRNAs not annotated as conserved in the database were then BLAST against the nucleotide database in NCBI (https://www.ncbi.nlm.nhi.gov) to search for similar sequences. We used human and mouse as the target organisms. The megablast algorithm was selected and all parameters were left to default values (Supplementary Data S8).

We retrieved the co-expression network for all the DElincRNAs with functional annotation in ZFLNCRNA (gene ontology and KEGG pathway) and classified the genes as modulated or unmodulated by comparing the obtained gene list to our previously reported differentially expressed mRNAs (GSE98539)^9^. Expressed genes were considered as modulated with FDR < 0.05, whereas genes with FDR > 0.05 were registered as unmodulated (Supplementary Data S7). Later, gene ontology and pathway analyses on the generated gene sets were performed using WebGestalt (http://www.webgestalt.org)^67^ based on over-representation analysis. Only GO terms within the biological process category and pathways from KEGG were considered. DIANA-lncBase (v2)^50^ was used to search for experimentally confirmed miRNA-lncRNA interactions using the experimental module.

### RT-qPCR validation

RT-qPCR was performed to validate the expression level of 9 (random selected) out of 22 annotated DEmiRNAs. For cDNA synthesis, 1 μg of total RNA from each sample was used as input material (miScript II RT kit, Qiagen, USA). Following manufacturer’s instruction, 4 μL of 5 × miScript HiSpec Buffer, 2 μL of 10 × nucleotide triphosphate mix, 2 μL miScript reverse transcriptase mix, and 10 μL of RNAse free water, were mixed to reach a total volume of 20 μL. Reactions were incubated first for 60 min at 37 °C followed by 5 min at 95 °C to inactivate the reverse transcriptase.

qPCR (miScript PCR system, Qiagen, USA) reactions were set up in a total volume of 25 μL containing 12.5 μL 2 × QuantiTec SYBR Green PCR Master Mix, 2.5 μL 10 × miScript Universal Primer, 2.5 μL miRNA forward primer (5 mmol/L), 2.5 μL template cDNA (diluted ten times in RNAse free water) and 5 μL of RNAse free water. The polymerase was activated during 15 min at 95 °C followed by a 3-step amplification program as follows: denaturation 15 s 94 °C, annealing 30 s 57 °C, extension 30 s 70 °C. Finally, 95 °C 10 s, 65 °C 60 s, and 97 °C 1 s were used to obtain the dissociation curves. The ramp rate was adjusted to 1 °C/s. Measurements were performed in a LightCycler96 (Roche Diagnostics, Switzerland) from three biological replicates per experimental group (F_1_-γ and F_1_-C). Linreg (v2017.0)^68^ was used to determine primers efficiency and to calculate the expression level of miRNAs as measured by the threshold cycle values (Ct). Quantification values derived from five technical replicate per biological replicate. Relative quantification was normalized to a synthetic spike-in kanamycin RNA and expression levels were determined as equation 2^−ΔΔCT^. Forward primers were designed with CLC-Workbench v6.0 (Qiagen, USA) (Table 8).

**Table 8:** miRNA primers used for RT-qPCR validation (CLC-Workbench v6.0).

miRNA	Primer sequence 5′- 3’	RT-qPCR	RNA-seq
dre-miR-150-5p	TCTCCCAATCCTTGTACCA	0.15	1.20
dre-miR-135-5p	TATGGCTTTCTATTCCTATGTG	0.33	1.16
dre-miR-204-5p	TTCCCTTTGTCATCCTATGC	0.16	0.97
dre-miR-let7j-5p	TGAGGTAGTTGTTTGTACAG	0.60	0.72
dre-miR-141-3p	AACACTGTCTGGTAACGA	− 0.41	− 0.80
dre-miR-2189-3p	TGATTGTTTGTATCAGCTGTGT	− 1.32	− 1.32
dre-miR-205-5p	TCCTTCATTCCACCGGAGTCT	− 1.22	− 1.82
dre-miR-716-3p	AACGAGAGCTTTGAAGGCC	− 0.29	− 1.00
dre-miR-738-3p	GCTACGGCCCGCGTCGGGA	− 1.12	− 3.42

RT-qPCR values are given as log2 fold change (FC) of the expression in generation F_1_ from exposed parents (F_1_-γ) relative to generation F_1_ from control parents (F_1_-C). RNA-seq values are given as log2 FC and derived from differential expression analysis using edgeR (v3.24.3, Bioconductor) by pairwise comparison between F_1_-γ and F_1_-C group. Spearman’s correlation coefficient = 0.7667 (p = 0.02, confidence interval 95%, alfa < 0.05).

The obtained mean relative miRNA expression values (F_1_-γ vs F_1_-C) were compared to mean relative miRNA expression values for the same miRNAs from RNA-seq, and a Spearman’s correlation coefficient was calculated (p < 0.05) (Graphpad Prism 7 v7.0, La Jolla, USA).

## Supplementary Information

Supplementary Figures

Supplementary Data S1

Supplementary Data S2

Supplementary Data S3

Supplementary Data S4

Supplementary Data S5

Supplementary Data S6

Supplementary Data S7

Supplementary Data S8

## Data Availability

Sequencing and expression data is available at https://www.ncbi.nlm.nih.gov/geo/query/acc.cgi?acc=GSE152189.
