# Supplementary material for: Altered non-coding RNA expression profile in F1 progeny 1 year after parental irradiation is linked to adverse effects in zebrafish
Source: Sci Rep. 2021 Feb 18;11:4142. doi: 10.1038/s41598-021-83345-3 (PMC7893006; doi:10.1038/s41598-021-83345-3)
Supplement: Supplementary file 1 — Supplementary Figures [file 41598_2021_83345_MOESM1_ESM.pdf]

Altered non-coding RNA expression profile in F_1 progeny one year after parental irradiation is linked to adverse effects in zebrafish

Leonardo Martín, Jorke H Kamstra, Selma Hurem, Leif C Lindeman, Dag A Brede, Håvard Aanes, Igor Babiak, Amilcar Arenal, Deborah Oughton, Brit Salbu, Jan Ludvig Lyche, Peter Aleström.

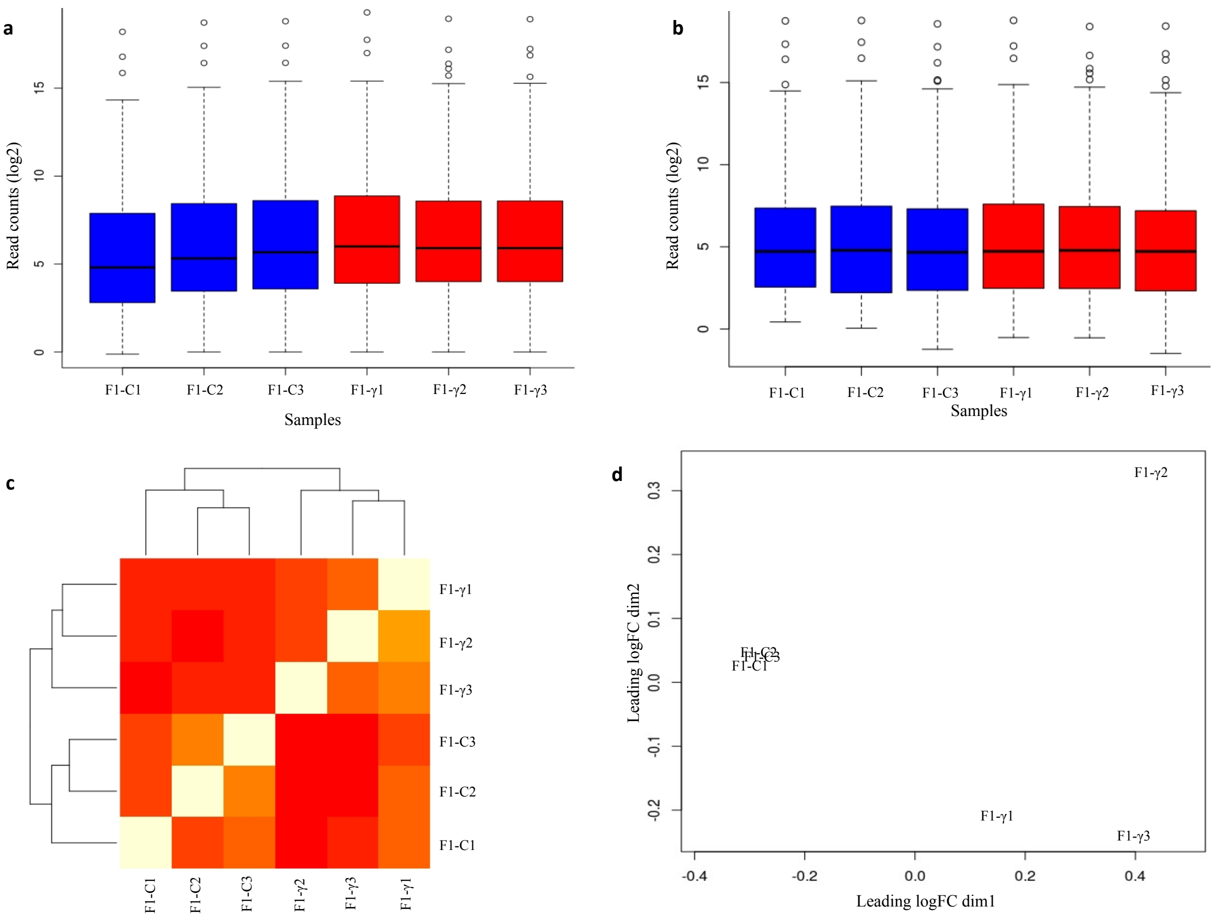

Supplementary Figure S1. Statistical exploration of miRNA expression dataset in generation F_1 from parents exposed to 8.7 mGy/h γ-radiation (F_1-γ) and F_1 from control parents (F_1-C) (n = 3). Median comparison before normalization A). After TMM normalization: Median comparison B). Heatmap of Pearson's correlation coefficient C), and Multidimensional scaling plot D).

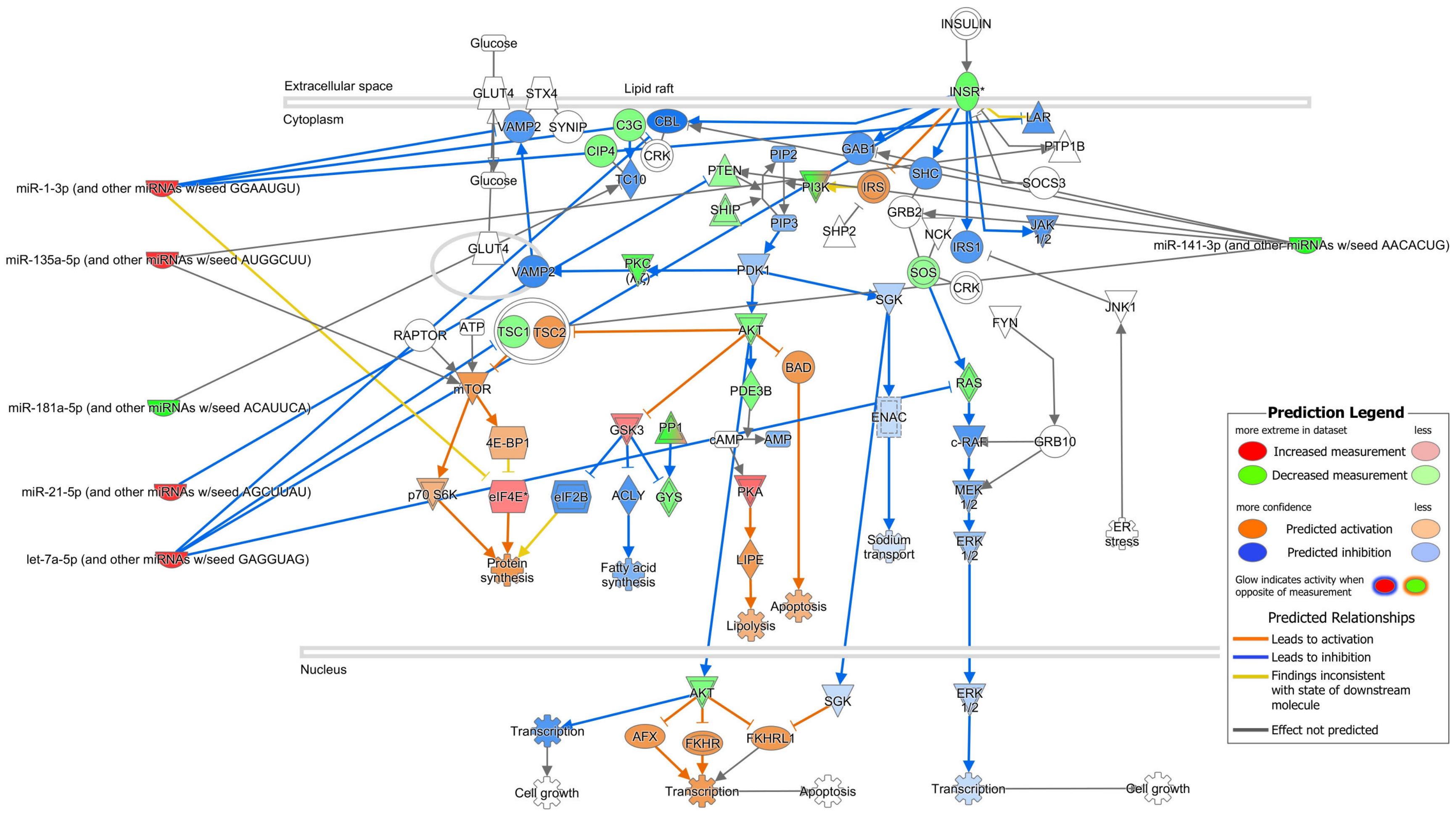

Supplementary Figure S2: Pathway analysis including molecule activity prediction, showing the insulin receptor-signaling pathway, with added miRNAs involved in the different nodes.

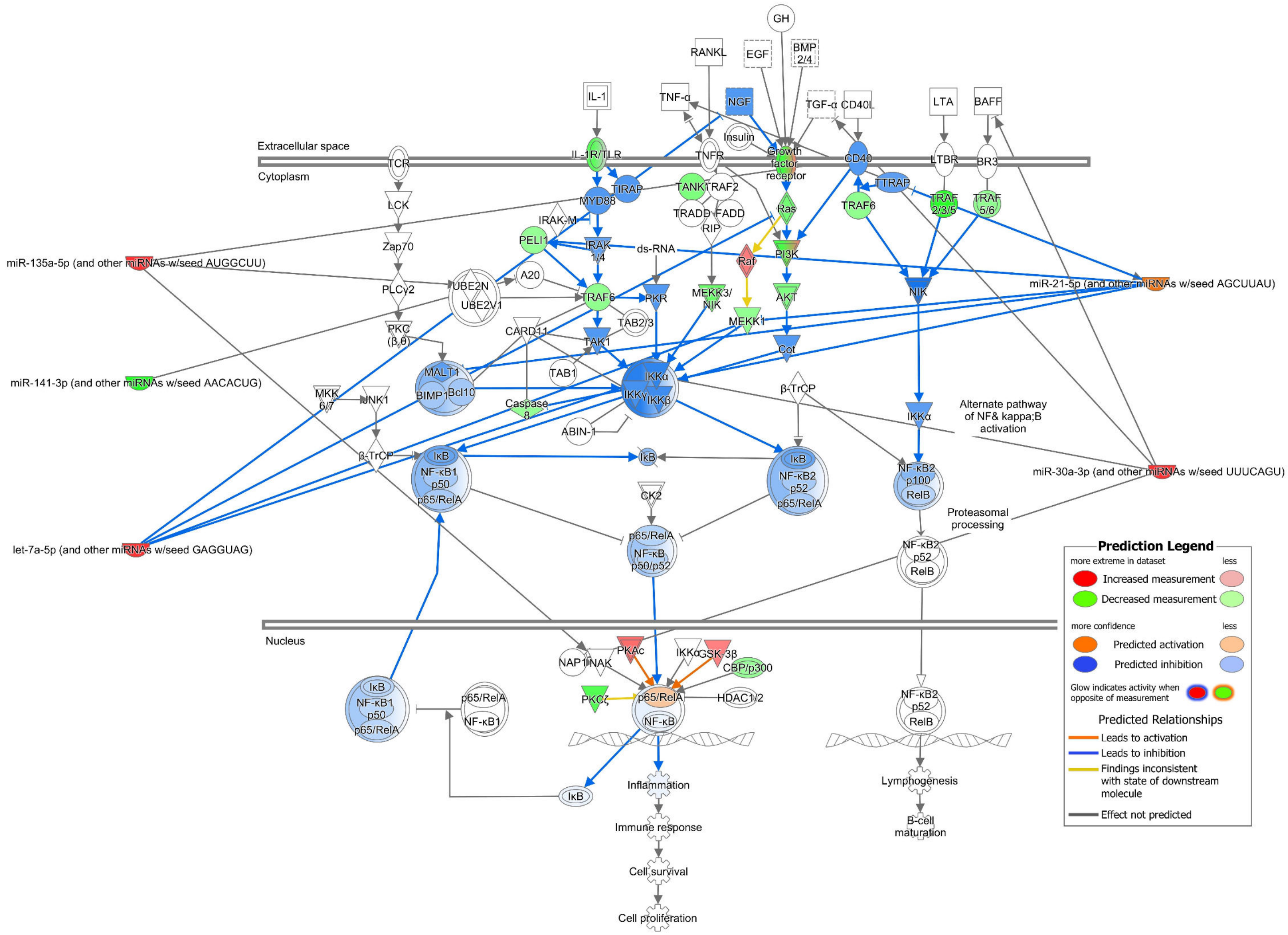

Supplementary Figure S3: Pathway analysis including molecule activity prediction, showning the NFkB signaling pathway, with added miRNAs involved in the different nodes.

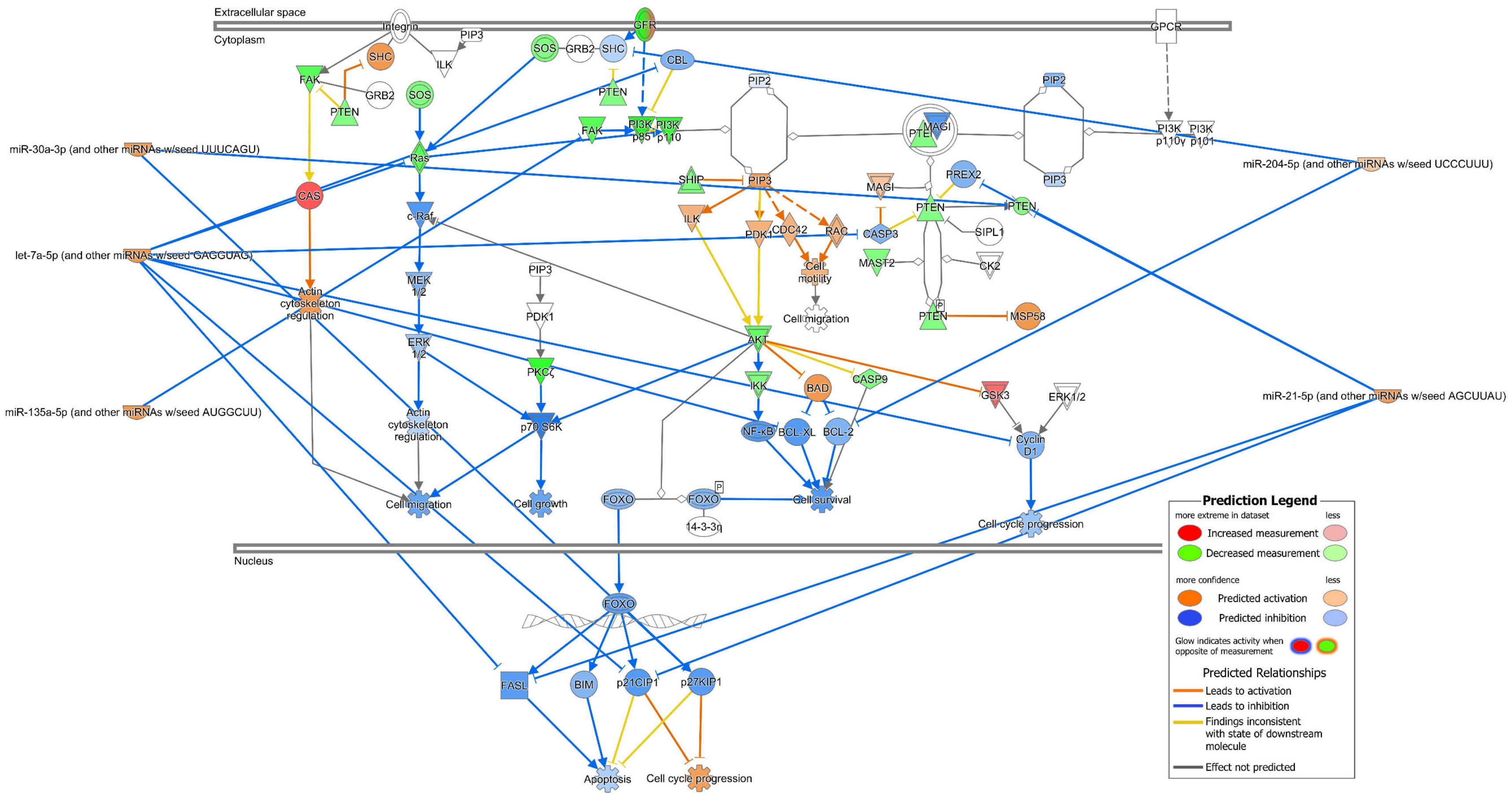

Supplementary Figure S4: Pathway analysis including molecule activity prediction, showing the PTEN signaling pathway, with added miRNAs involved in the different nodes.

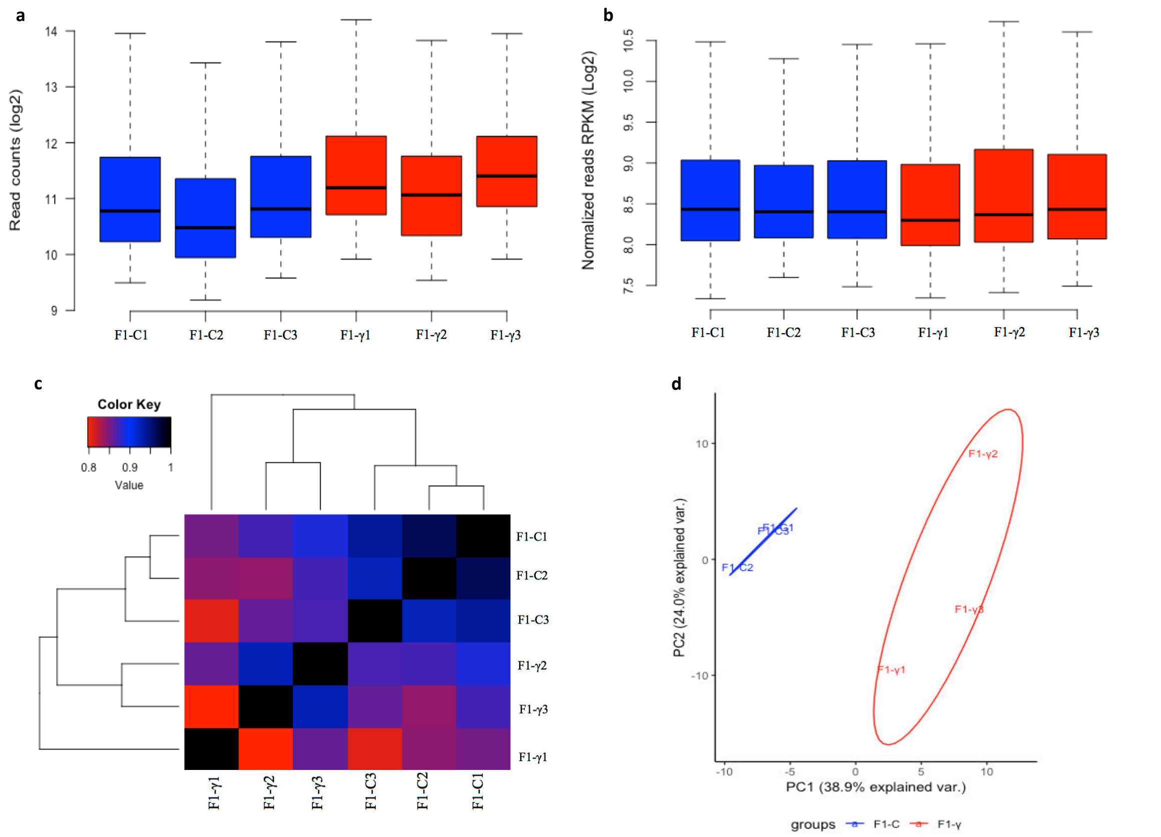

Supplementary Figure S5. Statistical exploration of piRNA clusters expression in generation F_1 from parents exposed to 8.7 mGy/h γ-radiation (F_1-γ) and F_1 from control parents (F_1-C) (n = 3). A) Median comparison before normalization. After RPKM normalization: Median comparison (B). Heatmap of Pearson's correlation coefficient (C), and Principal Component Analysis (D).

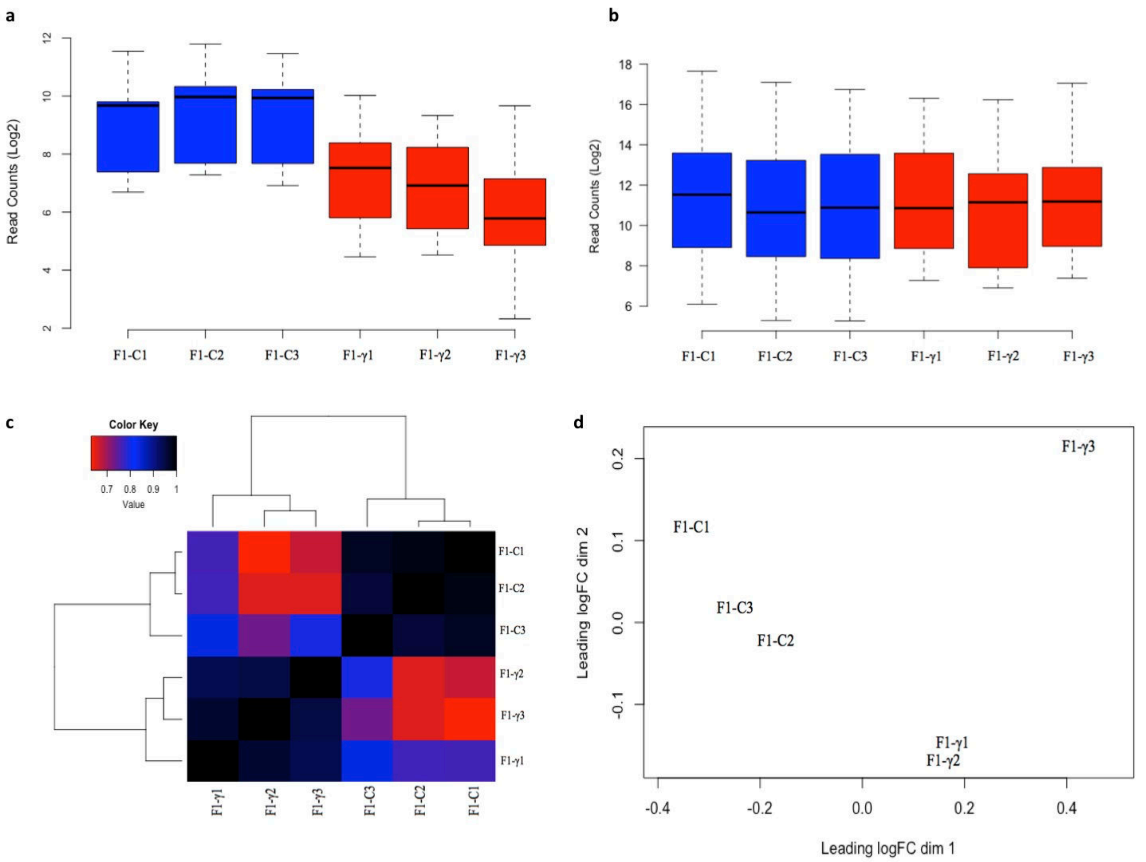

Supplementary Figure S6. Statistical exploration of snRNA expression in generation F_1 from parents exposed to 8.7 mGy/h γ-radiation (F_1-γ) and F_1 from control parents (F_1-C) (n = 3). A) Median comparison before normalization. After TMM normalization: Median comparison (B). Heatmap of Pearson's correlation coefficient (C), Multi-dimensional scaling plot (D).

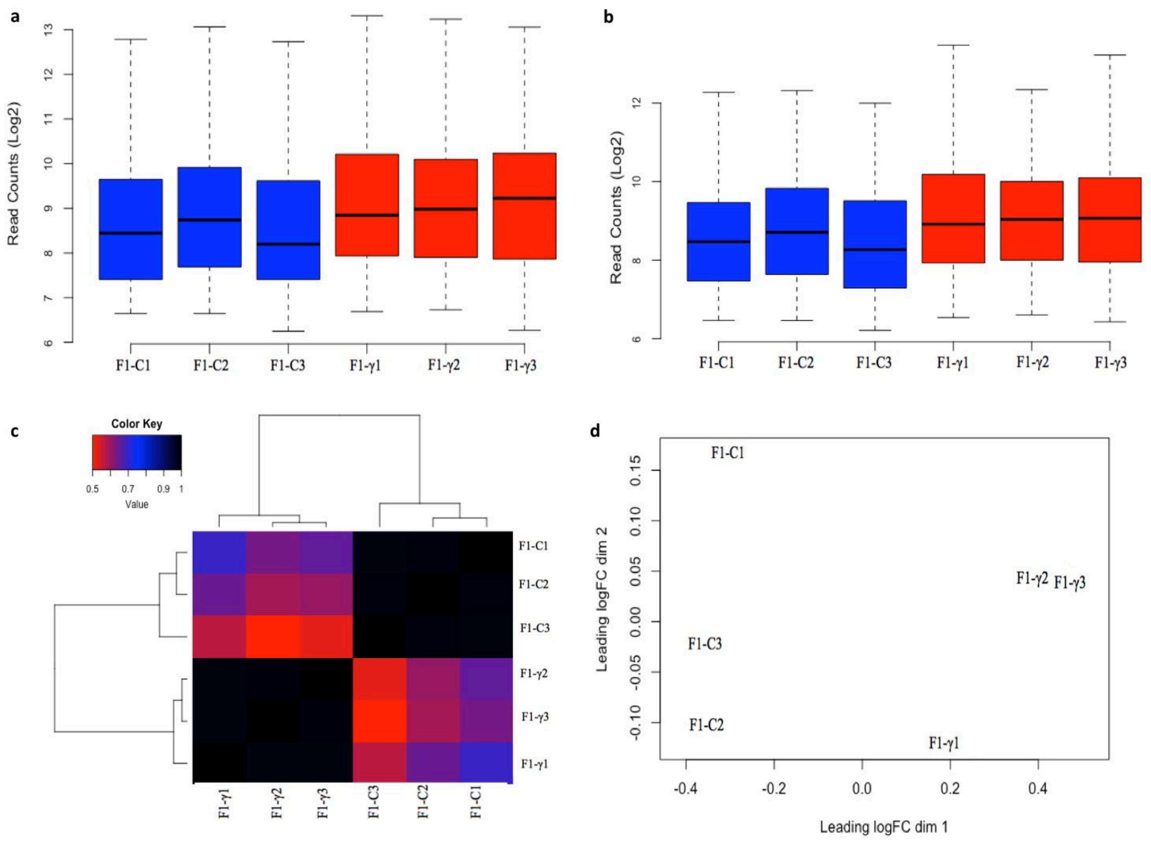

Supplementary Figure S7. Statistical exploration of lincRNA expression in generation F_1 from parents exposed to 8.7 mGy/h γ-radiation (F_1-γ) and F_1 from control parents (F_1-C) (n = 3). A) Median comparison before normalization. After TMM normalization: Median comparison (B). Heatmap of Pearson's correlation coefficient (C), Multi-dimensional scaling plot (D).

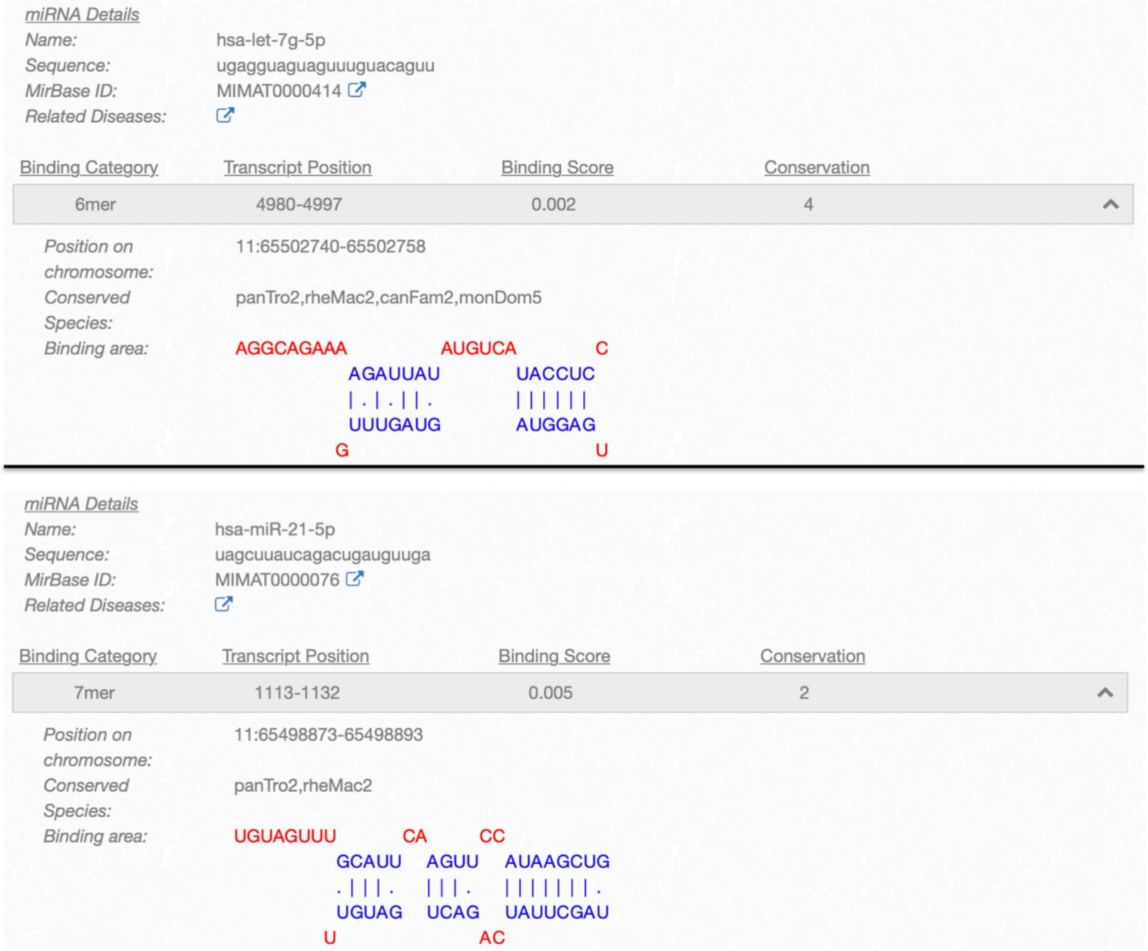

Supplementary Figure S8. Predicted interaction of hsa-let-7g-5p (top) and hsa-miR-21-5p (bottom) orthologs of dre-let-7g-5p and dre-miR-21-1-3p respectively, with MALAT1 (ENST00000534336). DIANA-lncBase (v2).
